# The rat frontal orienting field dynamically encodes value for economic decisions under risk

**DOI:** 10.1038/s41593-023-01461-x

**Published:** 2023-10-19

**Authors:** Chaofei Bao, Xiaoyue Zhu, Joshua Mōller-Mara, Jingjie Li, Sylvain Dubroqua, Jeffrey C. Erlich

**Affiliations:** 1 NYU-ECNU Institute of Brain and Cognitive Science at NYU Shanghai, Shanghai, China; 2 NYU Shanghai, Shanghai, China; 3 Shanghai Key Laboratory of Brain Functional Genomics (Ministry of Education), East China Normal University, Shanghai, China; 4 Sainsbury Wellcome Centre, University College London, London, UK

**Keywords:** Decision, Dynamical systems, Psychology, Neural decoding

## Abstract

Frontal and parietal cortex are implicated in economic decision-making, but their causal roles are untested. Here we silenced the frontal orienting field (FOF) and posterior parietal cortex (PPC) while rats chose between a cued lottery and a small stable surebet. PPC inactivations produced minimal short-lived effects. FOF inactivations reliably reduced lottery choices. A mixed-agent model of choice indicated that silencing the FOF caused a change in the curvature of the rats’ utility function (*U* = *V*^*ρ*^). Consistent with this finding, single-neuron and population analyses of neural activity confirmed that the FOF encodes the lottery value on each trial. A dynamical model, which accounts for electrophysiological and silencing results, suggests that the FOF represents the current lottery value to compare against the remembered surebet value. These results demonstrate that the FOF is a critical node in the neural circuit for the dynamic representation of action values for choice under risk.

## Abstract (web summary)

This study examined the role of rat frontal and parietal cortices in choosing whether to gamble versus play it safe. A combination of perturbations, electrophysiology and quantitative modeling establishes that the frontal cortex is important for representing the expected utility of options in the service of economic choice.

## Main

**Extended Data Fig. 1:**
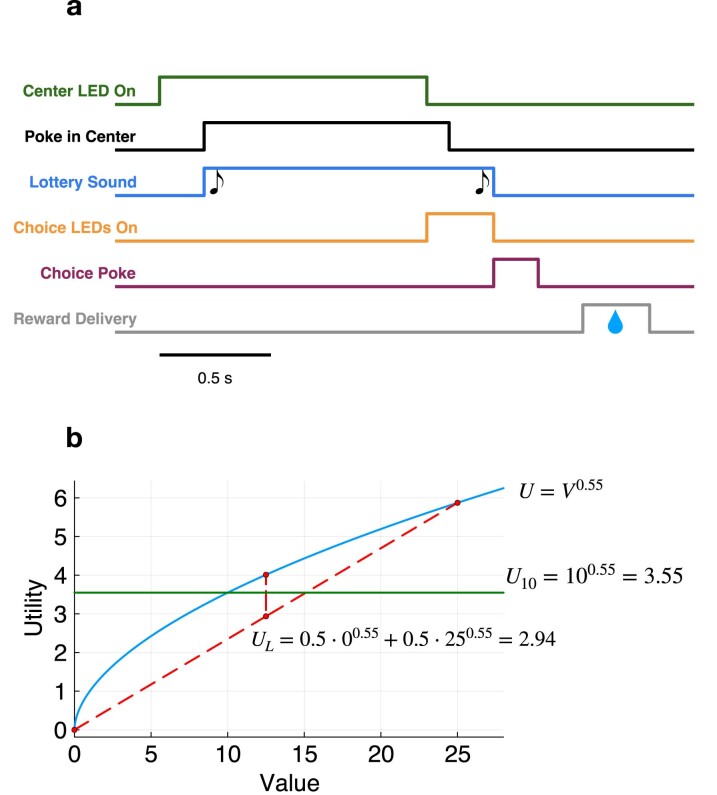
Timeline of the risky-choice task and the relationship between utility curvature and risk aversion. **a**. Illustration of the timeline of a risky-choice trial. **b**. The relationship between utility curvature and risk aversion. Consider a subject with a concave utility function, *U* = *V*^0.55^. They are offered a choice between a surebet of 10 dollars or a 50/50 lottery that pays out 25 dollars or nothing. The green line indicates the utility of the surebet *U*_*SB*_ = 10^0.55^ ≈ 3.55. The red dashed line connects the two possible outcomes of the lottery, 0^0.55^ = 0 and 25^0.55^ ≈ 5.87. The expected utility of the lottery is the weighted sum of the offers, *U*_*L*_ = 0.5 ⋅ 0 + 0.5 ⋅ 5.87 = 2.94. Since *U*_*L*_ < *U*_*SB*_ the subject will choose the surebet. If this lottery was offered 100 times, the subject would have 1000 dollars total, instead of close to 1250 dollars if they had chosen the lottery each time.

Understanding decisions under risk is crucial for public health. Excessive risk-taking relates to addiction and dangerous behaviors^1^, whereas inadequate risk-taking leads to missed opportunities—a rat will not thrive if it is unwilling to risk predation to forage. Any avoidance of uncertainty, either in the laboratory or in real life, can be considered ‘risk aversion’, but such avoidance can come from distinct cognitive constructs^2^. In economics, the most common framework to explain risk aversion is expected utility theory^3^. The core idea of the theory is that external rewards (food, water or money) are converted into an internal subjective value or ‘utility’. The shape of the utility function influences risk preference. Subjects with more concave utility functions are more risk averse because, for concave functions, the mean of the utilities of two offers will be less than the utility of the mean of the offers (Extended Data Fig. 1b)^4^.

When using expected utility theory to understand risky decisions, there is an assumption that the subject understands the risks and gains associated with offers under consideration. With human subjects, offers can be verbally or visually indicated, making this assumption reasonable. With animal subjects, there is necessarily a process of learning the relationship between cues and outcomes through experience^5^. Because of this obstacle, attempts to link expected utility theory to the underlying neural mechanisms has mostly been done in humans and monkeys^6–9^ and only rarely in rodents^10^. These studies have found activity related to expected utility in regions typically associated with reward and value representation^9,11,12^ but also in regions associated with orienting decisions, including the parietal cortex^13^ and frontal cortex^14,15^, because subjects were typically asked to respond by shifting gaze to a target. To our knowledge, the correlational findings are supported by only a single causal study that found that silencing the supplementary eye field in frontal cortex shifted monkeys to be less risk seeking^16^.

Here we present results from a ‘risky choice’ task where rats make choices under ‘expected uncertainty’—that is, a well-known but stochastic environment^17,18^. On each trial, rats made decisions between a ‘surebet’ (small but guaranteed reward) and a lottery with fixed probability and cue-guided magnitude. Our model-based quantification of animals’ behavior incorporated parameters to capture utility curvature, decision noise and choice biases. With this framework, we examined the causal contribution of the frontal orienting field (FOF) in the frontal cortex and the posterior parietal cortex (PPC). Both of these areas have been implicated in perceptual decision-making in rodents but with important distinctions. First, perturbations of the FOF consistently influence perceptual decisions^19–21^, whereas PPC perturbations have less reliable effects^19,22–24^. Second, it has been argued that the representation in the FOF is ‘post-decision’—that it represents animalsʼ current choice rather than a continuum of evidence^20,25^—whereas the PPC directly encodes the momentary evidence^20,22^. Based on studies of the role of the frontal-parietal network in economic decisions and rodent studies of FOF and PPC in perceptual decisions, we hypothesized that silencing the FOF would disrupt choices in a stimulus-independent manner (that is, increase biases) and that silencing the PPC might bias choices in a stimulus-dependent manner, corresponding to its linear encoding of subjective value^13^ and perceptual decision variables^20^.

We found that silencing PPC had a small short-lived effect on decisions under risk (but biased free choice). Surprisingly, we found that silencing of FOF made rats risk averse. Model-based analysis of these results indicated that the risk aversion was caused by an increase of the concavity of the utility function. Moreover, this effect was parsimoniously explained by a dynamical model where the FOF is part of a network for encoding the utility of the lottery on each trial. This dynamical model predicted that the FOF should contain neurons that monotonically increase with the lottery magnitude. To test this, we recorded neurons in the FOF and found neurons positively correlated with lottery value. Moreover, the magnitude of the lottery could be accurately decoded from FOF population activity. Together, these results suggest that the FOF is a key node in a network for representing the expected utility of options in the service of economic choice.

## Results

### Task and behavior

**Fig. 1:**
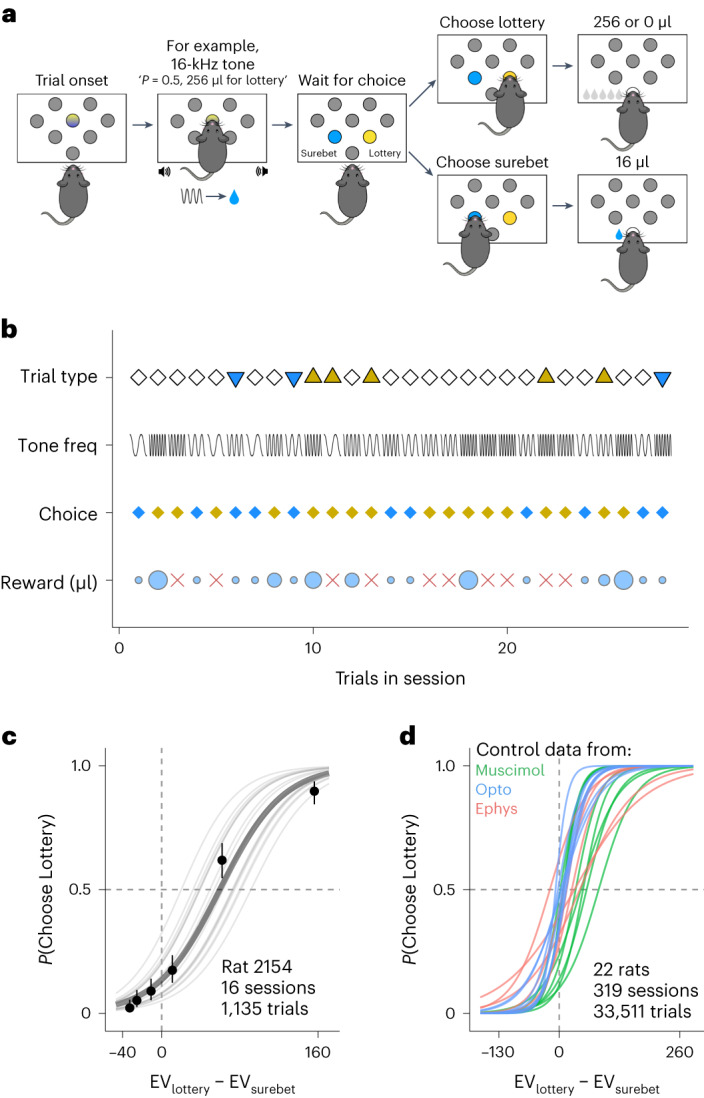
The task and animal behavior. **a**, Schematic of the task. On each trial, rats initiated the trial by fixating in the center port. At the onset of fixation, a tone was played, indicating the magnitude of the lottery. The tone remained on until the response in the choice port (see Methods for details). **b**, An example sequence of trials. For trial type, white diamonds, yellow triangles and blue triangles represent choice trials, forced lottery trials and forced surebet trials, respectively. The sine waves in the ‘tone freq’ illustrate that the lottery sound varied from trial to trial. Animalsʼ responses are marked in diamonds, with yellow for lottery and blue for surebet. The reward received (μl) on each trial is shown in light blue circles, whose size represents the relative amount. The red cross indicates a ‘lottery-lose’ trial. **c**, Example subject performance from rat 2154 (from the muscimol experiments). The probability of choosing lottery is plotted as a function of the EV of lottery minus the EV of surebet in μl of water. The circles with error bars are the mean and 95% binomial CIs. The lines are psychometric curves generated by a logistic fit; the thin gray lines are fits to each session; and the thick gray line is the fit to all the sessions combined (*n* = 1,135 trials, 16 sessions). **d**, Subject performance from control sessions for all experiments. For muscimol animals, control sessions were 1 d before an infusion event. For optogenetic animals, we include the control trials from sessions with laser stimulation. The lines are logistic fits from each animal, with the color of the line indicating the experiment that animal participated in (*n* =33,511 trials, 319 sessions, 8 rats for infusion, 8 rats for optogenetics and 6 rats for electrophysiology).

We trained rats on a risky choice task where they chose between a lottery and a surebet choice on each trial. The value of the lottery on each trial was indicated by an auditory cue (Fig. 1a,b). In this paper, we present behavior only from sessions after the animals recovered from surgery (for implantation of cannulae, fibers or electrodes). Unless otherwise specified, control trials for muscimol experiments came from the sessions from the day before the infusion sessions. For optogenetics animals, the control trials were no-laser trials from the same sessions as laser stimulation trials. Animals’ choices were largely consistent with a utility-maximizing strategy: they had relatively few violations of first-order stochastic dominance (that is, they chose the surebet when the lottery magnitude was less than the surebet magnitude), and they increased the proportion of lottery choices monotonically with increasing expected value (EV) (Fig. 1c,d). Visual inspection of the psychometric curves shows that most rats (19/22) were risk averse. That is, the point of subjective equality between the lottery and the surebet was when the EV of the lottery was greater than the surebet (Fig. 1d).

### Effects of silencing FOF and PPC

**Fig. 2:**
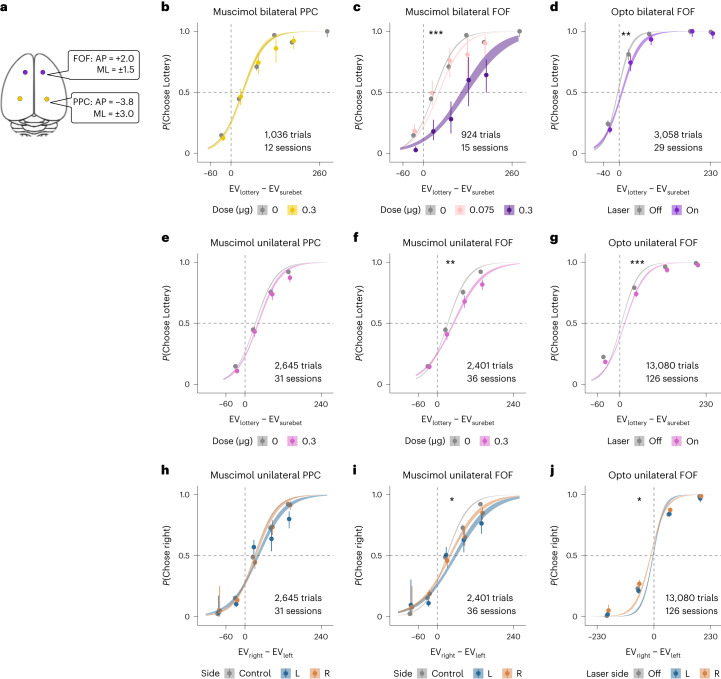
Effects of silencing PPC and FOF. **a**, Top-down view of the rat cortex with the target coordinates of FOF and PPC, where the cannulae or optical fibers were implanted. **b**–**g**, Probability of choosing the lottery given the difference in the EV of the lottery and the surebet. The circles with error bars are the mean and 95% binomial CIs. Points are jittered to avoid visual overlap of error bars. The ribbons are from a logistic fit to the data. The number of trials indicated on each panel are the infusion trials only. For panels showing data from muscimol experiments, the control data are from 1 d before any infusion. For panels with optogenetic (opto) silencing, the control data are the no-laser trials in the same sessions. Significance (one-sided, *P < 0.05, **P < 0.005, ***P < 0.0005) was based on an LR test between a mixed-effect model with and without a variable indicating which sessions (or trials) were drug/laser versus control. See the statistical appendix for details. **b**, Bilateral PPC infusions. Control sessions are in gray (*n* = 24 sessions, 7 rats), and 0.3 μg per side bilateral PPC infusions (*n* = 12 sessions, 7 rats) are in yellow (LR test, χ^2^(2, *N =* 9,501) = 3.15, *P* = 0.21). **c**, Bilateral FOF infusions. Control sessions are in gray (*n* = 17 sessions, 8 rats); 0.075 μg per side bilateral FOF infusions (*n* = 6 sessions, 5 rats) are in pink; and 0.3 μg per side bilateral FOF infusions (*n* = 9 sessions, 8 rats) are in purple (LR test, *χ*^2^(2, *N* = 9,389) = 16.43, *P* = 2.7 × 10^−4^). **d**, Bilateral optogenetic silencing of the FOF. Control trials are in gray, and opto silencing trials are in purple (*n* = 29 sessions, 5 rats, LR test, χ^2^(2, *N =* 3,085) = 11.42, *P* = 3.3 × 10^−3^). **e**, Unilateral PPC infusions. Control sessions (*n* = 100 sessions, 8 rats) are in gray, and 0.3 μg PPC infusions (*n* = 31 sessions, 8 rats) are in pink (LR test, χ^2^(2, *N =* 11,110) = 3.67, *P* = 0.16). **f**, Unilateral FOF infusions. Control sessions (*n* = 39 sessions, 8 rats) are in gray, and 0.3 μg unilateral FOF infusion sessions (*n* = 36 sessions, 8 rats) are in pink (LR test, χ^2^(2, *N =* 10,866) = 13.52, *P* = 1.2 × 10^−3^). **g**, Unilateral optogenetic silencing of the FOF (left FOF *n* = 75 sessions, 8 rats; right FOF *n* = 51 sessions, 7 rats; LR test, χ^2^(2, *N =* 13,080) = 25.84, *P* = 2.4 × 10^−6^). **h**–**j**, These panels contain the same data as **e**–**g** but are re-organized to show possible ipsi-contra biases. The *x* axes are the EV of the right option versus the EV of the left option; the *y* axes are the probability of choosing the right option. If unilateral silencing produced contralateral neglect, then we would expect the psychometric curves from right infusions (in brown) to be above the left infusions (in dark blue) (**h**: *n* = 100 control sessions, 12 sessions for left PPC, 19 sessions for right PPC, 8 rats. LR test, χ^2^(1, *N =* 2,645) = 1.18, *P* = 0.28. **i**: *n* = 100 control sessions, 16 sessions for left FOF, 20 sessions for right FOF, 8 rats. LR test, χ^2^(1, *N =* 2,401) = 6.63, *P* = 0.01. **j**: *n* = 75 sessions for left FOF, 51 sessions for right FOF, 8 rats. LR test, χ^2^(1, *N =* 4,658) = 4.59, *P* = 0.03).

We first used pharmacological silencing to test the causal role of the FOF and the PPC in the task. All muscimol animals experienced three different types of inactivations (left, right and bilateral) in two brain areas (FOF and PPC) (Fig. 2a). In total, we include 7,456 choice trials from 127 infusions sessions into the FOF and PPC of eight rats. The details of region, order and dosage of the infusions for each rat are shown in Supplementary Fig. 2. We followed up the muscimol experiments with optogenetic silencing of the FOF with halorhodopsin. In those experiments, we performed left, right and bilateral inactivations. We used generalized linear mixed-effects models (GLMMs) to test the effects of perturbations in two ways. First, we examined whether bilateral and unilateral silencing led to changes in ‘risk preference’: the probability of choosing the lottery given *EV*_*lottery*_ − *EV*_*surebet*_. Second, we tested whether the probability of choosing the right option given *EV*_*right*_ − *EV*_*left*_ was affected by unilateral silencing, as ‘contralateral neglect’ is commonly observed with unilateral impairment of the frontal-parietal network^19,26^. Details of the GLMM results can be found in the statistical appendix. The *P* values reported in this section are based on likelihood ratio (LR) tests between mixed-effect models with and without a variable indicating which sessions (or trials) were drug (or laser) versus control. In other words, the *P* value indicates whether a significant amount of the variance in the data is accounted for by the manipulation. We describe model-based analyses, which provide more insight into the nature of the deficits induced by perturbations, in a subsequent section.

**Extended Data Fig. 2:**
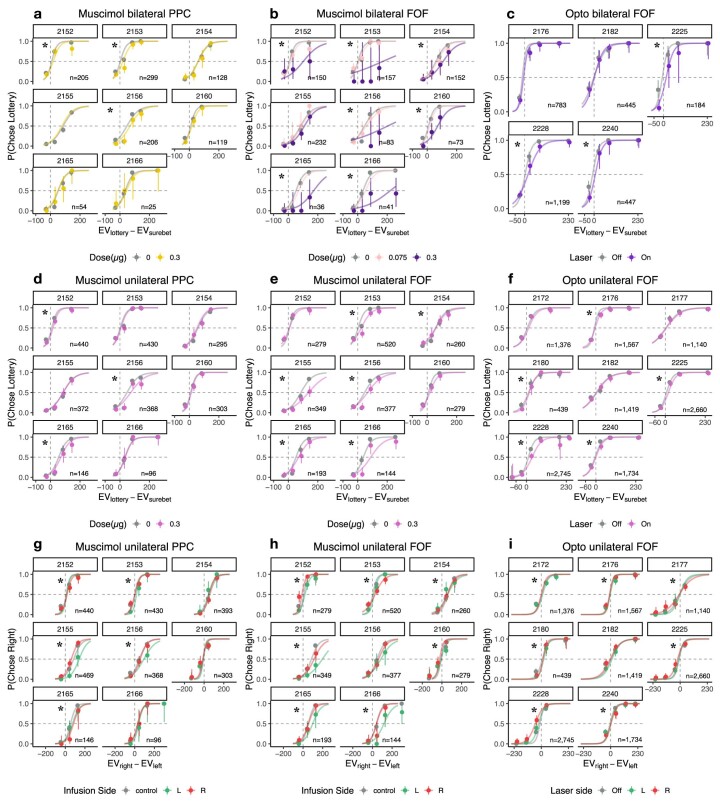
Inactivations by subject. The circles with error bars are the binned mean and 95% binomial confidence intervals. The lines are the model predictions generated by a mixed-effects model. Significance was tested with a likelihood ratio test between logistic fits with and without indicators of whether the data came from inactivation vs. control sessions (*χ*^2^ test, one-sided, * *p* < 0.05). Note: significance does not indicate that the direction of the effect for that subject was consistent with the population-wide effect. The direction of the effect can be inferred from the points. Note, statistics for the optogenetics animals included a within-session factor which is difficult to visualize in the by-subject plot (Individual trial number shows in the plots). **a**. Bilateral PPC muscimol inactivations (n = 1,036 trials, 7 rats). **b**. Bilateral FOF muscimol inactivations (n = 924 trials, 8 rats). **c**. Bilateral FOF optogenetic inactivations (n = 3,058 trials, 5 rats). **d**. Unilateral PPC muscimol inactivations (n = 2,645 trials, 8 rats). **e**. Unilateral FOF muscimol inactivations (n = 2,401 trials, 8 rats). **f**. Unilateral FOF optogenetic inactivations (n = 13,080 trials, 8 rats). **g,h,i**. Same data as in d,e,f but organised to show left-right biases rather than lottery-surebet biases.

Bilateral silencing of the PPC did not significantly influence risk preference (*P* = 0.207; Fig. 2b)—an effect consistent in five of eight animals (Extended Data Fig. 2a). Likewise, unilateral PPC infusions did not significantly alter risk preference (*P* = 0.160; Fig. 2e) nor did they cause contralateral neglect (*P* = 0.277; Fig. 2h). We also observed no reliable effect on reaction time (Supplementary Fig. 4). It was recently found that the behavioral effects of silencing the PPC can be short-lived^26^. We tested whether a similar phenomenon might be at play by excluding data in each PPC session after a certain number of trials. We found that, with a cutoff of 60 trials or fewer (for example, analyzing only the first 45 trials in each session), there is a significant effect of silencing PPC: animals became more risk averse (*P* = 0.017; Extended Data Fig. 8b).

Bilateral silencing of the FOF resulted in substantial reduction in lottery choices (*P* = 0.0003; Fig. 2c). Results from seven of eight subjects were consistent with this (Extended Data Fig. 2b). The mean indifference point (in units of *EV*_*lottery*_ − *EV*_*surebet*_ = μl of water) shifted from 50.9 ± 11.6 in control to 154.4 ± 23.5 under 0.3 μg of muscimol (*t*_8_ = − 3.95, *P* < 0.001). In other words, inactivating bilateral FOF was equivalent to adding around 100 μl to the surebet. Bilateral silencing of the FOF did not consistently change animals’ reaction time (*P* = 0.764). However, there was a significant slowing effect in four animals (Supplementary Fig. 4c), possibly due to muscimol spillover into the adjacent M1 (Supplementary Fig. 3a). Overall, the slowing effect from bilateral FOF inactivation was less reliable across animals than the effect on choice (compare Extended Data Fig. 2b with Supplementary Fig. 4c), suggesting that the effect on choice was not primarily driven by changes in movement. Results of bilateral optogenetic silencing of the FOF were consistent with, although smaller than, the muscimol effects: choices shifted away from the lottery (*P* = 0.003; Fig. 2d) without any effect on reaction time (*β*_*opto*_ = 0.026 ± 0.018, *P* = 0.068; compare Extended Data Fig. 2c with Supplementary Fig. 4e).

Unilateral muscimol infusions into the FOF caused a small but significant reduction in lottery choices (*P* = 0.001; Fig. 2f) without a significant change in reaction time (*P* = 0.06; Supplementary Fig. 4d). The reduction in lottery choices was observed in six of eight rats (Extended Data Fig. 2e). Unilateral optogenetic silencing produced a similar effect (*P* < 0.001; Fig. 2g). When examined from the perspective of contralateral neglect, there was a small significant effect of both pharmacological (*P* = 0.010; Fig. 2i) and optogenetic (*P* = 0.032; Fig. 2j) silencing. Note that the muscimol experiments were not well counterbalanced: seven of eight rats had the lottery on the right. Thus, the observation that, on average, both left and right infusions had fewer rightward choices could be due to this experimental limitation. That said, the left infusions shifted choices more to the left than the right infusions, and the optogenetic rats were better counterbalanced. The ipsi-contra effects were surprisingly weak compared to the large ipsilateral biases caused by unilateral FOF silencing in previous tasks^19,21,27^. We think that this difference is due to task differences. Previous tasks, with large contralateral impairments, had a short-term memory requirement for successful performance, whereas the risky choice task does not have one: the lottery sound played until the subject responded.

### A three-agent mixture model of risky choice

**Fig. 3:**
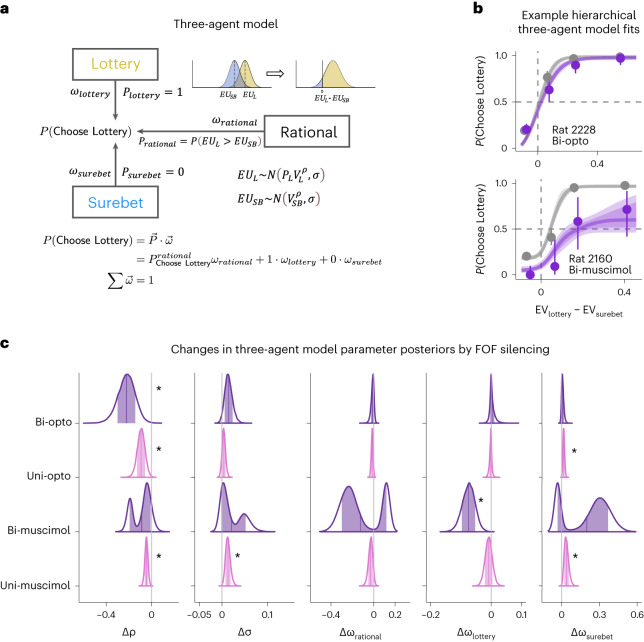
FOF inactivation reduced the exponent of the utility function. **a**, The three-agent model of risky choice. **b**, Psychometric curves from two example animals. The circles with error bars are the binned mean and 95% binomial CIs. The ribbons are model predictions generated using the fitted parameters (control, gray; bilateral FOF inactivation, purple). The solid line represents the model-predicted probability of lottery choice, and the dark and light shades represent 50% and 80% CIs, respectively. Similar plots for all animals are shown in Extended Data Figs. 3 and 4 (*n* = 801 control trials and 417 opto trials for rat 2228; *n* = 782 control trials and 73 infusion trials for rat 2160). **c**, Posterior distributions of changes in model parameters due to FOF silencing. An asterisk indicates that 97.5% of the posterior was not overlapping with 0. Silencing FOF consistently increased risk aversion through reducing the curvature of the utility function, *ρ*.

Although the GLMM analyses are effective for detecting whether a certain perturbation influenced behavior in the task, they do not provide insight into the specific role that the brain region might play. To better understand the task behavior and the effect of perturbations, we developed a three-agent mixture model (Fig. 3a). The first agent is a ‘rational’ utility-maximizing agent^3^ with two parameters: *ρ*, which controls the curvature of the utility function (*U* = *V*^*ρ*^), and *σ*, which captures the decision noise. For the rational agent, *ρ* controls the risk preference and the indifference point on the psychometric curve. If *ρ* < 1, then an agent is risk averse; if *ρ* > 1, the agent is risk seeking. The other two agents are stimulus-independent agents that habitually choose either the lottery or the surebet. The relative influence of the agents is controlled by their mixing weights *ω*, where $\sum \overrightarrow{\omega }=1$. The choice on each trial is, thus, a weighted outcome of the ‘votes’ of three agents, each implementing a different strategy (equation (41)). We estimated the joint posterior over the parameters for all subjects using Hamiltonian Monte Carlo sampling of a hierarchical Bayesian model in Stan^28,29^ and validated that the model can correctly recover generative parameters from synthetic data (Extended Data Fig. 5a). Details of the modeling, including the priors, can be found in Methods. The motivation for developing the mixture model was that the animals’ choices, although clearly sensitive to the lottery offer, showed some stimulus-independent biases. In other words, even for the best lottery, they sometimes chose the surebet, and, for the worst lottery (which had a value of 0), they sometimes chose the lottery. For example, subject 2160 has a psychometric curve that asymptotes in a way that is inconsistent with a pure utility-maximizing strategy (Fig. 3b, gray): even when the lottery is worth nothing, 2160 chooses the lottery about 18% of the time. Moreover, previous work has suggested that silencing the FOF can produce a stimulus-independent bias^19,25^, so it was important to include this in the model to account for that effect in perturbation experiments.

Trial history effects could have been incorporated by allowing model parameters to vary depending on the outcome of the previous trial, as in ref. ^18^. However, our animals seemed to understand that the lottery offer was independent across trials, and there were no statistically significant effects of a previous trial’s outcome on choice in control sessions (GLMM, *β*_*lottery*−*win*_ = 0.20 ± 0.12, *P* = 0.08; *β*_*lottery*−*lose*_ = 0.17 ± 0.09, *P* = 0.08). Earlier in training, these same animals did show trial history effects, which diminished with sufficient training. For this reason, we formulated the three-agent mixture model without trial history parameters. Our animals’ behavior stood in contrast to a substantial number of published results demonstrating strong trial history effects in rodent decision-making even when the optimal strategy is to use information only on the current trial^18,30^. We speculate that an important difference is that, in traditional rodent two-alternative forced-choice tasks, the rewards were delivered at the choice ports, but, in our task, all rewards were delivered at a single reward port. When rewards are delivered at the choice ports, then a positive association may be formed at the port, which could influence choices on subsequent trials^31^.

**Extended Data Fig. 3:**
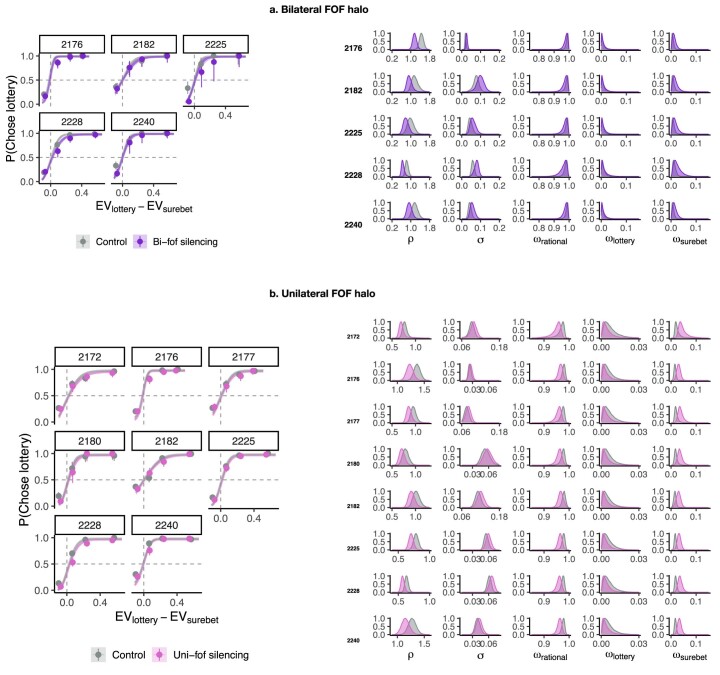
Individual subject fits in optogenetics experiments. Note: the posterior density plots (right panels) show posteriors for control and silencing data, but the samples are in fact paired. Thus, the overlap of the distributions does not reflect the statistical estimate of the shift. See Supplementary Tables 1 and 2 for the confidence intervals of the parameter shifts. **a**. Left: Subjects’ choices superimposed with the inactivation model fit on control (in gray) and bilateral FOF inactivation (in purple) dataset simultaneously. The circles with error bars are the binned mean and 95% binomial confidence intervals. The ribbons are model predictions generated using the fitted parameters. The solid line represents the model-predicted probability of lottery choice, the dark and light shade represent 50%, 80% confidence intervals, respectively. Right: Posterior distributions of transformed model parameters for each subject in the bilateral FOF optogenetic experiments (n = 3,058 trials, 5 rats). **b**. as in **a** but for the unilateral optogenetic silencing of FOF (n = 13,080 trials, 8 rats).

**Extended Data Fig. 4:**
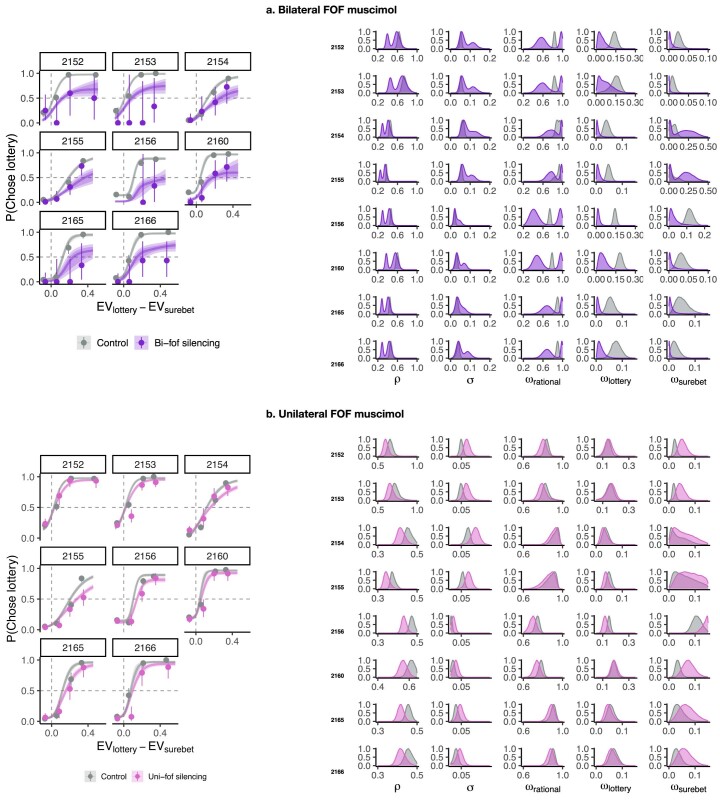
Individual subject fits in FOF muscimol experiments. Note: the posterior density plots (right panels) show posteriors for control and silencing data, but the samples are in fact paired. Thus, the overlap of the distributions does not reflect the statistical estimate of the shift. See Supplementary Table 3 for the confidence intervals of the parameter shifts. **a**. Left: Subjects’ choices superimposed with the inactivation model fit on control (in gray) and bilateral FOF muscimol (in purple) dataset simultaneously. The circles with error bars are the binned mean and 95% binomial confidence intervals. The ribbons are model predictions generated using the fitted parameters. The solid line represents the model-predicted probability of lottery choice, the dark and light shade represent 50%, 80% confidence intervals, respectively. Right: Posterior distributions of transformed model parameters for each subject in the bilateral FOF muscimol experiments (n = 924 trials, 8 rats). **b**. as in **a** but for the unilateral muscimol silencing of FOF (n = 2,401 trials, 8 rats).

The three-agent model fit the control behavior well (see two example animals in Fig. 3b, gray; all animals in Extended Data Figs. 3 and 4). All animals in the infusion experiments and four of eight animals in the optogenetic experiment had decelerating utility functions (median *ρ* < 1; Supplementary Tables 1–3). Note that the ‘effective’ risk preference is influenced by both *ρ* and *ω*. For example, the indifference point of rat 2152 is close to 0, implying that it is effectively risk neutral (Extended Data Fig. 4a). However, this comes from its bias toward choosing the lottery (*ω*_*lottery*_ = 0.14), balancing its decelerating utility function (*ρ* = 0.64; Supplementary Table 3). The animals had small but varying levels of decision noise (*σ* = 0.05 (0.04, 0.06), mean and 95% confidence interval (CI) of posteriors across animals), indicating that they were sensitive to water rewards just a few μl apart. Their choices were guided mostly by the rational agent (*ω*_*rational*_ = 0.84 (0.79, 0.88)), with little influence from the lottery agent (*ω*_*lottery*_ = 0.14 (0.10, 0.18)) and the surebet agent (*ω*_*surebet*_ = 0.03 (0.01, 0.04)).

To quantify how the perturbations influenced model parameters, we designed the three-agent model to be ‘doubly’ hierarchical: we fit all subjects simultaneously and also fit control and perturbation experiments simultaneously. We fit a separate model for each perturbation experiment (six models—FOF:uni/bi x muscimol/opto; PPC:uni/bi). As with the GLMM analyses, for the muscimol fits, the same control data were re-used for all fits. For the optogenetic fits, control data were no-laser trials from the same sessions, so the control data for bilateral and unilateral optogenetics models were non-overlapping. We chose priors for the effects of perturbation such that the model favored no effect of inactivation (that is, zero mean).

PPC infusions of muscimol led to no reliable changes across subjects for all parameters, which was consistent with the results from the GLMM (Supplementary Table 3). However, we re-fit the model for just the first 40 trials of the PPC muscimol sessions and found that there were significant shifts in the mixing fraction, *ω*. In other words, the shift toward choosing the surebet was best explained by a change in stimulus-independent bias, not in the parameters of the rational agent (Extended Data Fig. 8c).

### FOF inactivation reduced the utility curvature

Across the four FOF silencing experiments, there was a consistent decrease in the curvature of the utility function, *ρ* (Fig. 3c, Δ*ρ* < 0). Bayesian statisticians generally discourage the use of *P* values, but we considered a shift to be statistically significant if 97.5% of the credible interval of the posterior did not overlap with 0 (a two-sided test). For three of four experiments, Δ*ρ* was significantly below 0. Only the bilateral muscimol inactivation of the FOF gave ambiguous results: the posterior of the shifts had two modes. One mode favored an interpretation of the data with a Δ*ρ* ≪ 0, and the other mode favored an interpretation with a decrease in the weight of the rational and lottery agents and substantial increases in the surebet agent. Note, the bilateral muscimol experiments also had the least amount of data. Besides the consistent effect on *ρ*, there was also a tendency to see an increase in the weight of the surebet agent, Δ*ω*_*surebet*_ > 0. This reached significance in both of the unilateral silencing experiments (Fig. 3c, pink).

**Fig. 4:**
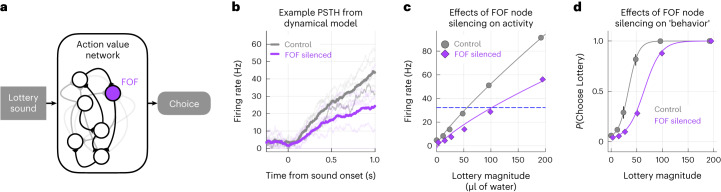
Dynamical model of FOF silencing. **a**, We implemented a six-node rate model of a distributed action value network with random connectivity (${W}_{ij} \sim {{{\mathcal{N}}}}(5/6,1)$). The FOF was one of the six nodes (in purple). The input to the network was the lottery magnitude. **b**, Example of the network response to lottery sound with magnitude of 96 μl under control conditions (with all the nodes active, in gray) and under FOF silencing (the FOF node is set to zero, in purple). The dark traces represent the mean network activity, and the light traces represent the activity of the six individual nodes. **c**, Silencing FOF scales down the representation of the action value of the lottery, which could explain the shift in *ρ*. We ran the network for 20 ‘trials’ of each lottery ∈ [0, 12, 24, 48, 96, 192] μl. The gray circles are the mean and 95% CI for the network response in the control conditions, and the purple diamonds are the mean and 95% CI for the network response when the FOF node is silenced. Fitting a power law utility function (*U* = *V*^*ρ*^) to the network activity gives *ρ* ≈ 0.76 for control and *ρ* ≈ 0.6 after FOF silencing. The thin lines are power law utility functions that approximate the transformation from units of reward (μl) to utils in spikes per second. **d**, Behavioral effects of the FOF silencing in **c**. The scaling of activity results in a stimulus-dependent shift, quantitatively similar to our experimental observations.

How could silencing the FOF change the exponent of the utility function? Previous silencing and modeling results suggested that the FOF is part (1/6) of a distributed circuit for maintaining a prospective memory of choice^27^. Inspired by that finding, we constructed a six-node rate model of a distributed circuit for encoding action value (or action utility), where the FOF represented one node in that network (Fig. 4a)^32^. Three nodes (not the FOF) received input representing the magnitude of the lottery. The all-to-all weight matrix was generated randomly, but the distribution of the weights was chosen such that the response of the network to the inputs was in the dynamic regime of the nodes (0 < *Hz* < 100). Other network parameters (noise *σ* and time-constant *τ*) were chosen to generate a control network response with reasonable dynamics (Fig. 4b) that encoded the lottery value in the population activity of the network (Fig. 4c, gray circles). In this regime, we found that silencing the FOF node scaled down the network’s responses. Note that the scaling is not a trivial 1/6 reduction in the average firing rate but reflects the contribution of the FOF node to the overall network dynamics (Fig. 4c; firing drops from 45 Hz to 22 Hz for the largest lottery). We can think of this network as encoding the expected utility of choosing the lottery by transforming the lottery sound into ‘utils’ (encoded as spike rate). At the time of the go-cue, this activity could become bistable—where the utility of the surebet determines the unstable fixed point^33^. Alternatively, a downstream region could compare the output of this network with the remembered surebet utility (denoted by the dashed blue line in Fig. 4c). In any case, scaling down the input–output transform of the network (Fig. 4c, purple circles) would shift the indifference point (the lottery that had the same activity level as the surebet comparator), which would, behaviorally, appear as a change in the power law utility function *U* = *V*^*ρ*^. For the control network, the network approximates a function with *ρ* ≈ 0.76. After silencing the FOF node, the exponent of the utility functions shifted down, *ρ* ≈ 0.6 (Fig. 4c), and resulted in a rightward stimulus-dependent shift in the psychometric curve (Fig. 4d). The dynamical model explains why silencing the FOF caused animals to reduce their lottery choices (Fig. 2c,d,f,g) through a change in the exponent of the utility function (Fig. 3c). This model is a substantial departure from previous ones where each hemisphere guides contralateral choices^25,27,34^. We implemented two versions of those models, one with the FOF as post-decision^20,25^ and one where the FOF encodes the value of the contralateral choice (Extended Data Fig. 6). Both models predicted larger biases for unilateral than bilateral inactivations and predicted that bilateral silencing would produce an increase in noise, not a shift away from the lottery. This argues that the role of the FOF in this task is distinct from the role it plays in motor planning for tasks that have a working memory component.

### Physiological evidence of value encoding in FOF

**Fig. 5:**
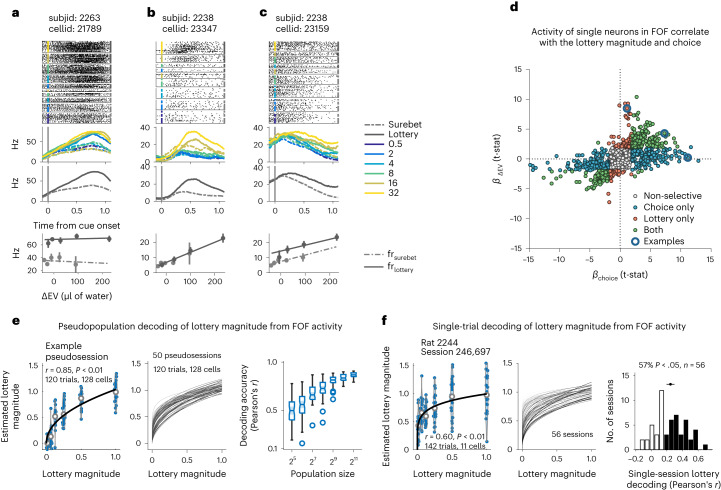
Neural activity in the FOF encoded lottery value and upcoming choice action during the fixation period. **a**–**c**, Example neurons. Spike raster plots and PSTHs of three representative neurons show heterogeneous dynamics during the fixation period. The rasters and PSTHs were aligned to the lottery cue onset and sorted by different lottery cues (indicated by different color) and upcoming choices (solid line for lottery choices, dashed line for surebet choices). The lower PSTH was sorted only on the basis of upcoming choices. The bottom row (firing rate versus Δ*EV*) summarizes the relationship among the neural activity, lottery value and choice. The lines are fits of a linear model (Hz ~ Δ*EV* * choice) to the data (dots with error bars represent mean ± s.e.m.; **a**: *n* = 150 trials, **b**: *n* = 106 trials, **c**: *n* = 104 trials). **d**, Distribution of coefficients of the choice against Δ*EV* from the mixed-effects linear models to characterize the individual neuronal responses to upcoming choices or lottery values. Of 1,690 recorded neurons, 23.5% (*n* = 393) were tuned for upcoming choice alone; 6.8% (*n* = 114) were tuned purely for the lottery values; and 18.3% (*n* = 309) were tuned for both. Blue circled data show where the three example neurons are located in the scatter plot. **e**,**f**, Pseudopopulation (**e**) and single-trial decoding (**f)** of lottery magnitude from FOF neural activities. Left panels show the examples of pseudosession and single session. The violins with the dot plots show the correlation between the cross-validated linear-model-estimated lottery magnitudes and the original lottery magnitudes (normalized for each session to the maximum magnitude). The solid lines are fits of a power law model $\hat{L}=\alpha {L}^{r}$, where the *L* is the normalized original lottery magnitudes, and $\hat{L}$ is the linear-model-estimated lottery magnitudes. Here, *r* captures the nonlinear relationship between the original and estimated lottery magnitude (**e**: for the example pseudosession, the Pearson’s correlation between the true value and power-law-model-estimated value, *n* = 120 trials from 128 cells *r* = 0.85, *P* = 5.5 × 10^−36^, two-sided, not adjusting for multiple comparisons. **f**: for the example session, the Pearson’s correlation between the true value and power-law-model-estimated value, *n* = 142 trials from 11 cells, *r* = 0.60, *P* = 7.6 × 10^−15^, two-sided, not adjusting for multiple comparisons). Middle panels show the power law fits for all pseudosessions with 128 cells and single sessions. The darkness of the lines show the scale of correlation between the original lottery magnitude and power-law-model-estimated ones (**e**: *n* = 50 pseudosessions, **f**: *n* = 56 sessions). Right panels shows the decoding accuracy for pseudopopulation and single-session decoding (**e**: *n* = 50 pseudosessions; the box-and-whisker plots show the median, lower/upper quartile, minimum/maximum and the outliers of the data, and the notch shows $median\pm (1.57\times interquartilerange)/\sqrt{n}$. **f**: *n* = 56 sessions; black bins indicate sessions that show significant decoding (Pearson’s correlation between the true value and power-law-model-estimated ones, *P* < 0.05). Black dot and bar above the histogram show the 95% CI of the mean).

The dynamical model (Fig. 4) suggested that neurons in the FOF should monotonically increase their firing rate with increased lottery values. To test this, we recorded single-unit activity from the FOF of six rats (Extended Data Fig. 7a). We found many neurons whose activity was consistent with value encoding in the service of decision-making (Fig. 5b–d) as predicted by our three-agent model of perturbations and our dynamical model. Specifically, during the fixation period, these neurons fired more on trials with higher lottery values, even when controlling for the choice of the animal (Fig. 5b,c). Many neurons also fired more for lottery choices than surebet choices, even when controlling for lottery magnitude (Fig. 5a,c,d). The presence of both pure lottery and pure choice neurons suggests that the FOF may contribute both to the representation of the value of options and also to the plan of future choice. This is consistent with the results from silencing: changes in both *ρ*, corresponding to representation of the lottery value, and *ω*, corresponding to representation of choice, were observed (Fig. 3c).

Of 1,690 neurons recorded, 423 (25.0%) significantly correlated with Δ*EV* (of these, 63.6% were positively correlated; the rest were negatively correlated) even when controlling for choice. We also found that, during fixation, the activity of 702 neurons (41.5%) predicted the upcoming choice of the animal (controlling for Δ*EV*), and 309 neurons (18.3%) significantly encoded both Δ*EV* and choice. To control for the possibility that the correlation with the lottery magnitude was, in fact, a correlation with the perceptual characteristics of the lottery sound, we recorded FOF neural responses to lottery sounds in animals that had not been trained on the task. In those recordings, we saw no more neurons with lottery ‘tuning’ than were expected by chance (Extended Data Fig. 7b).

To clearly establish that the lottery was encoded in the FOF neural activity, we performed cross-validated pseudopopulation decoding of the lottery magnitude (normalized by the maximum lottery in that session) using only trials where the subject chose the lottery. Even with pseudopopulations as small as 32 neurons (randomly selected regardless of their tuning), we could decode the lottery magnitude above chance (Fig. 5e; compare with shuffle in Extended Data Fig. 7c). Once we increased the pseudopopulation size to more than 200 neurons, decoding accuracy (Pearson’s *r*) was above 0.8. We can see that the lottery magnitude is not encoded linearly in the FOF: larger lottery magnitudes are scaled down, as might be expected from concave utility functions or Weber scaling. Together with our model-based analysis of perturbations and dynamical model, these analyses of neural activity strongly suggest that the activity in the FOF represents action values during planning as well as actions per se.

### Bilateral PPC inactivation did not impair learning

**Fig. 6:**
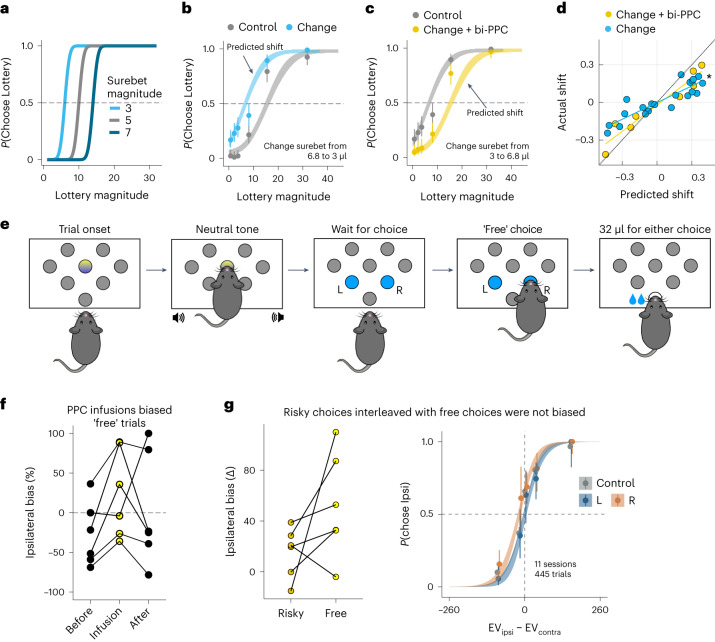
Infusions in PPC during surebet value change and free choice. **a**, Schematic showing that changing the surebet magnitude is equivalent to shifting the choice boundary. The data points were simulated from a risk-neutral agent using the three-agent model (*ρ* = 1, *σ* = 3, *ω*_*rational*_ = 1). A smaller surebet magnitude (light blue) horizontally shifts the psychometric curve leftwards; a larger surebet magnitude (dark blue) shifts the curve rightwards. The frequency-to-lottery mapping remains the same. **b**, Changing surebet magnitude from 6.8 to 3 shifted choices leftwards in one example animal. Combined trials from six sessions before the change are shown in gray, after the change shown in blue. One three-agent model was fit to all the trials, and the parameters were used for ribbon extrapolation (*n* = 547 trials for surebet magnitude is 6.8; *n* = 585 trials for surebet magnitude is 3; the circles with error bars are the mean and 95% binomial CIs). **c**, Same as **b** but with 0.6 μg per side bilateral PPC infusion, performed on the day of surebet change (from 3 to 6.8—*n* = 585 trials for surebet value is 3; *n* = 503 trials for surebet value is 6.8; the circles with error bars are the mean and 95% binomial CIs). **d**, The three-agent mixture model predicts the shifts in behavior well. One model was fit using all the sessions containing various surebet magnitudes for each animal. On the *x* axis is the predicted shift in probability choosing lottery: the difference in *P*(Choose Lottery) between model prediction using the new surebet magnitude and the session just before that change. On the *y* axis is the actual shift in *P*(Choose Lottery): the difference in *P*(Choose Lottery) between the first session of a surebet change and the session before that change. Sessions with just surebet change are in blue (*n* = 21, 4 animals); sessions with both surebet change and 0.6 μg per side bilateral PPC infusions are in gold (*n* = 8). There is a strong correlation between predicted and actual shift (Pearson’s correlation, *r* = 0.905, *P* = 1.6 × 10^−11^), and this relationship is significantly different between shifts where PPC was silenced and control shifts (LR test, χ^2^(2, 29) = 7.44, *P* = 6.4 × 10^−3^). **e**, Schematic of the free trials. After fixation at the center port accompanied by a neutral tone, the animal was free to choose the left or right port, both illuminated in blue LEDs. Choosing either port resulted in a reward twice the magnitude of surebet. The free trials were randomly interleaved with the forced and choice trials. **f**, Unilateral PPC infusions (0.6 μg) led to a significant ipsilateral bias toward the side of infusion. This panel shows % ipsilateral bias: (∑*choose*_*infusion*_*side* − ∑*choose*_*other*_*side*) / ∑*total*_*choices*, when the side of infusions was chosen to be the opposite to the animalsʼ preferred side. % ipsilateral bias was computed using free trials from the previous three sessions, the infusion session and the following three sessions for six subjects. **g**, Left: unilateral PPC infusions generated a significant 52 ± 16% (*t*(5) = 3.09, *P* = 0.027, mean ± s.e. across rats, *n* = 6, two-sided) change in % ipsilateral bias on free trials compared to control sessions (three pre-infusion sessions). For the choice trials from the same sessions, the change in % ipsilateral bias was not significant (15 ± 8%, *t*(5) = 1.92, *P* = 0.11, mean ± s.e. across rats, *n* = 6, two-sided). Right: performance on the choice trials was not affected. Control sessions from the three pre-infusion sessions (*n* = 65 sessions, 6 rats) are in gray; 0.6 μg left PPC infusions (*n* = 5 sessions) are in blue; and 0.6 μg right PPC infusions (*n* = 6 sessions) are in orange.

The GLMM analysis above shows that the rat PPC is not strictly necessary for the task: silencing PPC has an effect on behavior that is shorter than the pharmacological effect. However, numerous studies found that neural activity in PPC correlates with decision variables in both perceptual and economic tasks. The question thus remains: what is the purpose of these decision-related signals in PPC? Recently, Zhong et al.^24^ found that PPC silencing impaired the ability of mice to re-categorize previously experienced stimuli based on a new category boundary in an auditory decision-making task. Moreover, after the stimuli were re-categorized, PPC activity was no longer required for performance. Motivated by their findings, we tested whether PPC was necessary for re-categorizing stimuli in our task. To do so, we employed a model-based change in the surebet magnitude that effectively shifted the decision boundary without changing the frequency-to-lottery mapping (Fig. 6a). As such, some frequencies that had led to mostly lottery choices now led to mostly surebet choices (and vice versa, depending on the direction of the shift). To estimate the required shifts, we first fit the three-agent model on data from the past 14 sessions. We then used the fit to generate synthetic choices on different surebet magnitudes, until we found the one that resulted in a shift in the overall probability of choosing lottery (*P*(Choose Lottery)) close to the target (drawn uniformly from ±*U*(0.2, 0.3); see details in Methods). To familiarize animals with the new paradigm, their surebet magnitudes were changed weekly for 2 weeks. Two of six animals failed to show appropriate adaptation of behavior after change in surebet magnitude; they were excluded from analysis in this section. The other four animals reliably shifted their choices more toward surebet when its magnitude increased and more toward lottery when surebet magnitude decreased (see example animal in Fig. 6b, all other animals in Extended Data Fig. 8a).

After 2 weeks, on the day of surebet change, we infused 0.6 μg of muscimol into each side of the PPC in these four animals before the task. The animals learned the new surebet magnitude and adjusted their behavior in both control and PPC inactivation sessions (see example animal in Fig. 6c). To quantify the effectiveness of the surebet shift and the potential contribution of the PPC to the shift, we compared the predicted shifts to the actual shifts (Fig. 6d). Bilateral PPC inactivation did not impair the learning of new surebet magnitudes. In fact, we found the opposite. Normally, the actual shifts were smaller than the predicted shifts (Fig. 6d, blue). On days when the PPC was silenced, the actual shift was closer to the predicted shift (*β*_*predicted*_*shift*:*PPC*_ = 0.251 ± 0.091, *P* = 0.011; Fig. 6d, yellow). Thus, our results do not support the hypothesis that the PPC is required for shifting category boundaries—that is, categorizing a lottery as being better or worse than the surebet. Instead, the smaller-than-expected shifts (Fig. 6d, blue dots) could be explained as a contraction bias: the large shifts were unexpected given subjects’ prior beliefs about the magnitude of the surebet. Silencing the PPC (Fig. 6d, yellow dots) reduced this contraction bias—that is, weakened the influence of the prior on the behavior^35^.

### Unilateral PPC inactivation biases ‘free’ choices

To establish that our infusions into PPC were effective, after completing all of the experiments reported above, we added a ‘free’ trial type, as in ref. ^19^. On a free trial, both the surebet port and the lottery port were illuminated with blue LEDs after fixation, accompanied by a brief neutral tone. The animals were rewarded twice the magnitude of the surebet reward regardless of which port they chose (Fig. 6e). These types of trials have been demonstrated to be sensitive to unilateral silencing of the PPC^19,36^. We randomly intermixed 11% free trials with 22% forced trials and 67% choice trials on the control days. After a few sessions with the new trial type, rats expressed a consistent bias on the free trials and still performed the choice trials in a utility-maximizing way. The proportion of free trials was increased to 50% on the infusion day, with the rest being 12.5% forced trials and 37.5% choice trials. Infusions of muscimol (0.6 μg) into one hemifield of PPC (opposite to the animal’s preferred side) produced a substantial ipsilateral bias on free trials (Fig. 6f; *β*_*infusion*_ = 1.19 ± 0.50, *P* < 0.05). The ipsilateral bias in free trials was observed even while, consistent with our previous PPC inactivation results, there was no ipsilateral bias on the interleaved choice trials (Fig. 6g; *β*_*infusion*_ = 0.18 ± 0.14, *P* = 0.189). These free trial inactivation results provided a clear positive control for our PPC inactivations, demonstrating that the lack of effect on choice trials was not caused by a technical issue (such as clogged cannula).

## Discussion

We developed a risky choice task for rats where animals made cue-guided decisions between a lottery and a surebet on a trial-by-trial basis under expected uncertainty^37,38^. We developed a hierarchical Bayesian model to disentangle different elements of risk preference, including *ρ* as the exponent of the utility curve; $\overrightarrow{\omega }$ as the weights for rational, lottery and surebet agents; and *σ* for decision noise. We found that silencing the PPC resulted in a short-lived increase in risk aversion that was explained by changes in biases ($\overrightarrow{\omega }$). Additional experiments revealed that the PPC was not required for updating category boundaries but may play a role in representing a prior about the value of the surebet. Silencing the FOF resulted in a reliable increase in risk aversion that was explained by a decrease of the exponent, *ρ*, of the utility function. A dynamical model developed to understand the effects of silencing predicted that FOF would encode the lottery magnitude, which was confirmed by analyses of neural activity. Together, our results support a novel role for the FOF: representing the EV of actions.

The frontal and parietal cortices are strongly interconnected regions that work together to guide spatial attention and choice^39–41^. Although there is some consensus that the parietal cortex is more sensory^42^ and the frontal cortex is more motor^20,25,34,43^, many questions remain about their distinct contributions to different cognitive functions across species. The rodent FOF and PPC have been studied extensively in perceptual decision-making and motor planning, but, to our knowledge, this is the first study of their role in economic decision-making.

There are two opposing perspectives on the role of the FOF in decision-making. One is that the FOF is ‘post-decision’, and its main role is short-term memory for motor planning^25^. That is, when a choice requires integration, over time or over stimulus dimensions, that integration is done by an upstream region, which sends choice information to the FOF. This view comes from quantitative modeling of three lines of evidence: unilateral silencing of FOF produces vertical (stimulus-independent) shifts rather than horizontal (stimulus-dependent) shifts^19^; FOF seems to encode the sign of the decision variable^20^; and optogenetic silencing of the FOF during evidence accumulation influences the decision commitment but not the integration process^20^. The second perspective is that the FOF performs a broader role in cognition—for example, sensorimotor transformation^44^, multisensory integration^45^ and value coding^46,47^—as would be expected from a functional analog of the primate frontal eye field^21,40,48^. The frontal eye field is a key node in the neural circuit for goal-directed attention^41^, evidence accumulation^49,50^ and reward^15,51^, despite also being essential for short-term memory for motor planning^43^ and being very close to the motor output for shifting gaze^52^.

If the first view (FOF as post-decision) was correct, then we would have found only choice coding in FOF activity and stimulus-independent effects of silencing (Extended Data Fig. 6). In contrast, we found both choice and utility coding in FOF (Fig. 5) and caused stimulus-dependent changes with silencing (Fig. 3). Thus, our results support the second view. Moreover, there is a clear conceptual coupling between attention and utility: we naturally attend to objects with high utility. We also attend to objects with large negative utility (such as potential threats), which provides an interesting future opportunity for disentangling attention from utility. The dynamical model of our results (Fig. 4) suggests that the FOF represented the lottery and not the surebet, because the lottery was indicated by a cue on each trial, similar to findings in a simple cue–action association task^44^.

We speculate that the first view, FOF as post-decision, overweighted evidence from unilateral perturbation results and also depended too heavily on working memory as the essential cognitive construct. Here, by requiring animals to integrate probability with value, but not requiring integration across time (because our task has no working memory component), we revealed that the FOF is required for integration more generally, consistent with bilateral silencing results in accumulation of evidence^19^. Thus, it seems that the FOF participates in two distinct processes. For some task variables, such as the lottery EV, the information is distributed across the hemispheres (and potentially other brain regions), so unilateral silencing appears like a partial effect of bilateral silencing. For other variables, such as a motor plan in a two-alternative forced-choice task, the hemispheres compete rather than cooperate, so unilateral silencing generates a contralateral neglect (as in Extended Data Fig. 6). Further experimental and computational work is required to better understand when and how these distinct functions operate.

Activity in human and primate PPC has long been associated with decision variables in economic choice^9,13^ in addition to a broader role in attention and decision-making^41^. Thus far, we are not aware of any studies of the role of rodent PPC in economic decisions. PPC encodes task-related variables during perceptual decisions^20,39^, but its causal contribution remains elusive. Although our initial analyses led us to think that neither unilateral nor bilateral inactivation had a significant effect (as in evidence accumulation^19^), we were inspired by a recent finding^26^ to check if the behavioral effect of PPC inactivation might be short-lived. Indeed, we found that, early in sessions, silencing the PPC caused a stimulus-independent response bias toward the surebet. This finding should be interpreted with caution, because we searched for a definition of ‘early’ that would generate a significant effect. We chose not to perform optogenetic and electrophysiological recordings in the PPC because of our initial finding of nominal effects of muscimol. The discovery of a short-lived effect provides motivation to perform experiments that combine recordings and optogenetic perturbations across FOF and PPC (and downstream regions such as the superior colliculus) to potentially unravel the mystery of why there is this difference in the timescales of effects.

It has been argued that rodent PPC is important for visually guided decisions but not other modalities^22^. We previously suggested^19^ that, due to the anatomical proximity between the PPC and the visual areas, these inactivation results may be caused by spillover into the adjacent visual cortex. However, experiments using optogenetics have shown that targeted inactivation of PPC disrupted performance only on visual but not auditory processing^23,45^. As such, we cannot exclude the possibility that the short-lived effect on risky choice may be due to the modality of stimuli used. However, others have found that silencing the PPC impaired re-categorization of sounds^24^ and representation of sensory priors for sounds^35^, so the controversy over the modality-specific role of the PPC is not fully resolved. We tested both of these hypotheses by shifting the value of the surebet. We did not find evidence that the PPC is required for re-categorization. Rather, we found that animals had a contraction bias in these experiments, which was reduced by silencing the PPC (Fig. 6). This is consistent with the results from ref. ^35^ but in the domain of value rather than sounds. It may be that the rodent PPC contains distinct submodules, some of which are modality specific (that is, integration of visual evidence) and some of which are more general (that is, priors or trial history effects).

The neurobiology of risky choice in rodents has largely focused on systems and circuits classically involved in learning and reward: dopamine^11^, the amygdala^53,54^, basal ganglia^55,56^ and orbital-frontal cortex^57^. These studies often require subjects to choose between a stable surebet and a volatile uncertain option (although some have used cues to indicate lottery quality). These studies often find that changes in risk aversion due to perturbations are due to changes in ‘win–stay’ or ‘lose–switch’ strategies. The outcome of the previous trial did not significantly influence the choices of our subjects. Thus, our study has surprisingly little conceptual overlap with much of the existing rodent literature on risk^5^. Instead, our work is conceptually closer to monkey^16^ and human^9^ studies of risk. This underscores the importance of using behavioral tasks that are driven by theoretical frameworks to avoid confusion about the meaning of ‘risk’^38,58^. Only then can we make progress in disentangling the neural mechanisms underlying utility curvature, which dominates risk aversion under ‘expected uncertainty’, learning, which plays a substantial role in risk aversion under ‘unexpected uncertainty’^59^, and other cognitive processes^18,60–62^, which may contribute to risk aversion.

## Methods

### Subjects

A total of 26 rats (22 males, four females, between the ages of 2 months and 18 months) were used in this study, including 24 Sprague Dawley rats and two male Brown Norway rats (Vital River Laboratories). Animals were pair-housed during the training period and then single-housed after implantation. Of these 26 animals, six male Sprague Dawley rats and two male Brown Norway rats were used for the FOF/PPC muscimol inhibition experiment. These eight animals were placed on a controlled water schedule and had access to free water for 20 min each day in addition to the water they earned in the task. The other 18 Sprague Dawley rats (four females) were used for the in vivo electrophysiology recording and further optogenetic silencing of FOF experiment. For these animals, 4% citric acid water was available ad libitum in their home cage instead of controlled water access. All rats were kept on a reversed 12-h light/dark cycle and were trained during their dark cycle. Rats were handled and placed into experimental boxes by technicians who were blinded to the experimental goals and outcome assessments. Animal use procedures were approved by New York University Shanghai International Animal Care and Use Committee following both United States and Chinese regulations.

### Behavioral apparatus

Animal training took place in custom behavioral chambers located inside sound-attenuating and light-attenuating boxes. Each chamber (23 × 23 × 23 cm) was fit with eight nose ports arranged in four rows (Fig. 1a), with speakers located on the left and right side. Each nose port contained a pair of blue and a pair of yellow LEDs for delivering visual stimuli as well as an infrared LED and infrared phototransistor for detecting rats’ interactions with the port. The port in the bottom row contained a stainless steel tube for delivering water rewards. In the risky choice task, only four of the eight ports were used. Other tasks in the laboratory used all eight ports. The behavior task was controlled and acquired using Bpod (version 0.5, Sanworks) running on MATLAB 2018b (MathWorks). Each training session lasted for approximately 90 min.

### Behavior

Trials began with both yellow and blue LEDs turning on in the center port. This cued the animal to poke its nose into the center port and hold it there for 1 s, after which the center lights were turned off, and the choice ports became illuminated. We refer to this period as the ‘fixation’ period.

The animals for the muscimol infusion and optogenetics were allowed to withdraw briefly from the center port during fixation. If the animal poked into a different port other than the center port, a short white noise would play to indicate that this is a mistake. If the animal was out of the center port at the end of fixation, then we would wait until they returned before turning on the choice ports. They tended to withdraw after the initial poke but stayed close to the center port during the soft fixation period (Supplementary Fig. 1). The rats in the in vivo electrophysiological recording experiment were required to hold their noses in the center port during the entire fixation phase. If the rats failed to maintain the center poke during the fixation phase, this would count as a violation.

During the fixation period, a tone played from both speakers, indicating the lottery magnitude for that trial. Pure tone lottery cues were used for the muscimol infusion experiment, and clicks lottery cues were used for the optogenetic and in vivo electrophysiology recording experiment. For the pure tone lottery cues, there were six distinct frequencies indicating different lottery magnitudes (2.5– 20 kHz, 75 dB). The frequency of each lottery was around one octave away from the adjacent tones, making distinguishing the different offers perceptually easy^63^. For the clicks lottery cures, there were six distinct click frequencies (28, 45, 60, 81, 110 and 151 Hz). The individual clicks were short (3 ms) pure tones (10 kHz × lottery probability + 4 kHz). The six distinct lottery cues were randomly played in the final training phase. The cue frequency-to-lottery magnitude mapping and the location of the surebet port were counterbalanced across animals (Supplementary Table 5). At the end of fixation, the lottery port and the surebet port were illuminated with yellow and blue lights, respectively. The tone stopped as soon as the animal made a choice by poking into one of the choice ports. If the animal chose surebet, a small and guaranteed reward would be delivered at the reward port. If the animal chose lottery, based on the lottery probability, it would receive either the corresponding lottery magnitude or nothing. The lottery probability was titrated for each animal and ranged from 0.5 to 0.75 across all subjects. We refer to these trials as ‘choice’ trials. To ensure that the subjects experienced all the outcomes, the choice trials were randomly interleaved with trials that we refer to as ‘forced’ trials. The forced trials differ from choice trials in that only one of the two ports was illuminated and available for poking, forcing the animal to make that response. The forced surebet and forced lottery trials together accounted for 25% of the total trials. The inter-trial interval was between 3 s and 10 s (uniformly distributed). A trial was considered a violation if the animal failed to poke into the center port within 300 s from trial start or it did not make a choice 30 s after fixation. Violations were excluded from all analyses, except where they are specifically mentioned.

After all experiments presented in Figs. 2 and 6a–d were completed, as a positive control experiment for PPC inactivation, ‘free’ trials were introduced to six infusion animals. Free trials were similar to choice trials, and, at the end of fixation, both left and right ports were illuminated with blue LEDs. On free trials, a 1-kHz tone amplitude modulated at 4 Hz was played during fixation. The animal would receive a medium-sized reward (two times the surebet) regardless of which port it chose. The free trials were randomly interleaved with the choice and forced trials.

#### Training pipeline

Animal training took place in two distinct phases: the operant conditioning phase and the risky choice phase. In brief, in the operant conditioning phase, rats became familiar with the training apparatus and learned to poke into the reward port when illuminated with white LEDs. Trials began with the illumination of the reward port, and water reward was immediately delivered upon port entry. After the rats learned to poke in the reward port reliably, they proceeded to the next training stage where they were required to first poke into an illuminated choice port (left or right with blue lights, chosen randomly) before the reward port was illuminated for reward. They graduated to the risky choice phase if they correctly performed seven trials in a row.

In the risky choice phase, rats started with only two frequencies: the lowest (2.49 kHz for pure tone and 28 Hz for the clicks) and highest (19.91 kHz for the pure tone and 151 Hz for the clicks), corresponding to the smallest and largest lottery magnitude. Initially, there were more forced trials than choice trials to help them understand the task. Once the animals reliably differentiated between the low and high lottery choice trials, we increased the ratio of choice trials to force trials. Intermediate frequencies were added one by one, contingent upon good behavior in the choice trials with existing frequencies. The lottery probability and the surebet magnitude were adapted to each animal so that their preferences could be reliably estimated. For example, if an animal chose the lottery too often, the lottery probability would be decreased. The goal was to be able to accurately estimate parameters of the three-agent mixture model (described below). Once the animal reached stable performance on full six lottery frequencies, its lottery probability and surebet magnitude remained unchanged for the entire data collection period. The only exception was the surebet change experiment presented in Fig. 6a–d.

### Surgery

Surgical methods were similar to those described in ref. ^19^. The rats were anesthetized with isoflurane and placed in a stereotaxic apparatus (RWD Life Science). The scalp was shaved, washed with ethanol and iodopovidone and incised. Then, the skull was cleaned of tissue and blood. For cannula implantation, the stereotax was used to mark the locations of craniotomies for the left and right FOF and PPC relative to bregma on the skull. Four craniotomies and durotomies were performed, and the skull was coated with a thin layer of C&B Metabond (Parkell). Each guide cannula along with the injector (RWD Life Science) was inserted 1.5 mm into the cortex measured from the brain surface for each craniotomy. The guide cannulae were placed and secured to the skull one at a time with a small amount of Absolute Dentin (Parkell). The injector was removed from each guide once the guide was secured to the skull. After all four guide cannulae were in place, more Absolute Dentin was applied to cover the skull and further secure the guide cannulae. Vetbond (3M) was applied to glue the surrounding tissue to the Absolute Dentin.

The surgery for stereotaxic silicon probe implantations was similar to the surgery described in the cannula implantation. Here we only describe the procedures that were unique for this surgery. Ten rats were implanted with movable silicon probes (Cambridge NeuroTech) in either the left or right FOF for single-unit electrophysiology data collection. Six animals did the risky choice task; the other four animals were part of the lottery sound control experiment. Of the six risky choice rats, four were implanted contralaterally to the lottery side, and two were implanted ipsilaterally. The silicon probes were adhered to nano-drives (Cambridge NeuroTech) with super glue. Following the same procedure described above, we marked the location of the FOF (AP +2.5 mm and ML ±1.4 mm from bregma), and then a 1.5-mm craniotomy was drilled, followed by an entire dura resection. The craniotomy was then filled with saline-saturated Gelfoam to protect the brain tissue while the skull was coated with a thin layer of C&B Metabond and a 1–3-mm-high chimney built around the craniotomy using the Absolute Dentin. Then, the adjustable nano-drive assembled silicon probes were mounted to the stereotax. Ground wires were soldered to titanium ground screws located above primary visual cortex. The silicon probe was slowly lowered into the brain until all the recording sites were immersed into the tissue (1.3 mm DV for the H3 probes and 0.5 mm DV for the E probes). As FOF and PPC are on the dorsal surface of the cortex, craniotomy and durotomy were carefully executed to prevent any bleeding and brain tissue damage. The craniotomy was filled with Dura-Gel (Cambridge NeuroTech), and then the microdrive was cemented to the skull with Absolute Dentin.

For the optogenetic surgery, after the craniotomy and durotomy were made, 400 nl of adeno-associated virus (pAAV9-CamKII-eNpHR3.0-EYFP, about 5 × 10^12^ viral genomes per milliliter) were slowly injected into the FOF bilaterally using a glass needle micropipette, controlled by a nano-injector. The glass pipette tip was manually cut to ~30 μm diameter. To maximize the virus expression in the FOF, we performed the injection at different depth and tracts at each side of the FOF. At the targeted coordinates, an injection of 20 nl was made every 200 μm in depth starting from 200 μm below the brain surface until 1.5 mm. Four additional injection tracts were completed around the target coordinate, one each 500 μm anterior, posterior, medial and lateral from the central tract. For those tracts, an injection of 20 nl was made every 400 μm in depth starting from 400 μm below the brain surface until 1.5 mm. The injection speed was about 40 nl min^−1^. After the injection, the needle was maintained in the target area for at least 5 min to allow the virus to absorb, after which the needle was slowly withdrawn from the brain. Sharpened optic fibers (Plexon) were inserted 1.2 mm into the cortex measured from the brain surface for each hemisphere (2.5 mm rostral to bregma, 1.5 mm lateral to the midline and 1.2 mm depth with 10° angles). The craniotomy was sealed by Dura-Gel, and the optic fiber was secured to the skull with Superbond and Absolute Dentin.

The animals were individually housed after surgery and given 7 d to recover on free water before resuming training. eNpHR expression was allowed at least 4 weeks before testing for effects of optogenetic perturbation.

### Cannulae

All eight rats were implanted bilaterally in the FOF (AP +2 mm and ML ±1.5 mm from bregma) with 26 AWG guide cannulae (RWD Life Science) and in the lateral PPC (AP −3.8 mm and ML ±3.0 mm from bregma) with 26 AWG guide cannulae (four cannulae per rat total). The tip of the guide sat on the brain surface while the 33 AWG injector was extended 1.5 mm below the bottom of the guide cannula. Dummy cannula (which were left in the guides in between infusions) extended 0.5 mm past the guides into the cortex. Cannula placement was verified postmortem (Supplementary Fig. 3).

### Infusions

Infusions were performed once a week with normal training days taking place on all other days. This was to minimize adaptation to the effects of the muscimol and to have stable performance in the sessions immediately before infusion sessions. Animals were held by an experimenter during the infusion, and no general anesthetic was administered. On an infusion day, the rat was placed on the experimenter’s lap, and the dummy cannulae were gently removed and cleaned with iodine and alcohol and then rinsed in deionized water. The injector was inserted into the target guide cannula and reached 1.5 mm into cortex. A 1-μl syringe (Gaoge) connected via tubing filled with mineral oil to the injector was used to infuse 0.3 μl of muscimol into the cortex. The injection was done over 1 min, after which the injector was left in the brain for five more minutes to allow diffusion before removal. The thoroughly cleaned and rinsed dummies were placed into the guide cannula. The rats began training 2–53 min after the infusion; the average time between infusion and starting of the behavioral session was 27 min. The training was carried out by the technicians, who were blinded to the treatments. The complete list of all infusion doses, regions and order for each rat is provided in Supplementary Fig. 2.

### Optogenetics

After 4–6 weeks of viral expression, rats were first acclimatized to the optogenetic testing setup with the optical patch cable connected to the optical cannula on their head. The other end of the optical patch cables was connected to a fiber rotary joint (Newdoon) mounted on the ceiling of the sound attenuation chamber. After 2–3 d of acclimation with the setup, a 15–20-mW 532-nm laser (Aurora-300, Newdoon), triggered with a 5V TTL controlled by the Bpod system, delivered light through the fiber cable. Laser illumination occurred on 33% of trials (randomly interleaved). We performed entire trial silencing to see if optogenetic FOF perturbation led to the same results as muscimol infusion. A 3-s constant laser pulse was delivered to cover the entire trial. For the bilateral FOF silencing experiment, we used a fused splitter fiber patch cord (Newdoon) to evenly deliver the laser into both hemispheres. The left, right and bilateral FOF perturbation experiments were interleaved across sessions. The laser power was calibrated by a laser power meter (PM20A, Thorlabs) before and after the session.

### Behavioral data analysis

For all analyses, we excluded time-out violation trials (where the subjects disengaged from the ports for more than 30 s during the trial) and trials with reaction time longer than 3 s. For infusion animals, unless otherwise specified, the ‘control’ sessions refer to the sessions 1 d before any infusion event during the course of the experiment. For optogenetic analyses, control trials were the no-laser trials from the same sessions as a corresponding laser trial. As such, the control fits from unilateral opto can be different than from bilateral opto. Data analysis was not performed blinded to the conditions of the experiments. No statistical methods were used to pre-determine sample sizes, but our sample sizes are similar to those reported in previous publications^19,20^.

#### GLMMs

GLMMs were fit using the lme4 (version 1.1-29) R package^64^ and plotted using ggplot^65^. To test whether bilateral and unilateral muscimol infusions and opto perturbations had any effects on performance, we specified a mixed-effects model where the probability of a lottery choice was a logistic function of *EV*_*lottery*_ − *EV*_*surebet*_, treatments and their interaction as fixed effects. For the infusion experiment, the rat and an interaction of rat, *EV*_*lottery*_ − *EV*_*surebet*_ and treatments were modeled as within-subject random effects; the treatments were muscimol dosage (μg); and the control sessions were coded as a 0-μg dose. For the optogenetic experiment, the session and an interaction of session, *EV*_*lottery*_ − *EV*_*surebet*_ and treatments were modeled as within-session random effects, and the treatments were optogenetic stimulation (1 for optogenetic stimulation trials, 0 for control trials). The optogenetic stimulation trials were interleaved with the control trials within sessions. The EV of lottery is the product of the lottery probability and lottery magnitude (*EV*_*lottery*_ = *P*_*lottery*_ × *V*_*lottery*_). Similarly, *EV*_*surebet*_ denotes the EV of surebet, which is simply the value of surebet here (*EV*_*surebet*_ = *V*_*surebet*_, because *P*_*surebet*_ = 1). In GLMM formula syntax:

For the infusion experiment,

1 $${\mathsf{chose}}\_{\mathsf{lottery}} \sim {\mathsf{delta}}\_{\mathsf{EV}}* {\mathsf{treatments}}+({\mathsf{delta}}\_{\mathsf{EV}}* {\mathsf{treatments}}| {\mathsf{subjid}})$$

For the optogenetic experiment,

2 $${\mathsf{chose}}\_{\mathsf{lottery}} \sim {\mathsf{delta}}\_{\mathsf{EV}}* {\mathsf{treatments}}+({\mathsf{delta}}\_{\mathsf{EV}}* {\mathsf{treatments}}| {\mathsf{sessid}})$$

where chose_lottery is 1 if lottery was chosen on a trial; delta_EV is *EV*_*lottery*_ − *EV*_*surebet*_; subjid is the subject ID for each rat; and sessid is the session ID as factors. We regarded this model as the full model *mf*. To check perturbation effects on the animals’ risky choice performance, we drop the *treatments* from the fixed effects and fit a reduced model *mr* as follows:

For the infusion experiment,

3 $${\mathsf{chose}}\_{\mathsf{lottery}} \sim {\mathsf{delta}}\_{\mathsf{EV}}+({\mathsf{delta}}\_{\mathsf{EV}}* {\mathsf{treatments}}| {\mathsf{subjid}})$$

For the optogenetic experiment,

4 $${\mathsf{chose}}\_{\mathsf{lottery}} \sim {\mathsf{delta}}\_{\mathsf{EV}}+({\mathsf{delta}}\_{\mathsf{EV}}* {\mathsf{treatments}}| {\mathsf{sessid}})$$

The LR test was performed using lrtest(mf, mr) (from the lmtest R package) to determine whether the treatment had a significant effect on the risky choice performance.

To test whether unilateral infusions or optogenetic perturbation caused a left/right bias^19^, we specified a mixed-effects model similar to the one described above as the full model (*mf*):

For the infusion experiment,

5 $${\mathsf{chose}}\_{\mathsf{right}} \sim {\mathsf{rl}}\_{\mathsf{delta}}\_{\mathsf{EV}}* {\mathsf{treatments}}\_{\mathsf{side}}+({\mathsf{rl}}\_{\mathsf{delta}}\_{\mathsf{EV}}* {\mathsf{treatments}}\_{\mathsf{side}}| {\mathsf{subjid}})$$

For the optogenetic experiment,

6 $${\mathsf{chose}}\_{\mathsf{right}} \sim {\mathsf{rl}}\_{\mathsf{delta}}\_{\mathsf{EV}}* {\mathsf{treatments}}\_{\mathsf{side}}+({\mathsf{rl}}\_{\mathsf{delta}}\_{\mathsf{EV}}* {\mathsf{treatments}}\_{\mathsf{side}}| {\mathsf{sessid}})$$

where chose_right is 1 if the right port is chosen on this trial; rl_delta_EV is *EV*_*right*_ − *EV*_*left*_; and treatments_side is a categorical variable with three levels: left, right and control. The plots in Extended Data Fig. 2g–i show that the model fits for each rat, reflecting how the random effects allow for each rats’ data to be fit. To check whether the treatments had any effects on the animals’ left/right bias, we dropped the treatments_side in the fixed effects to fit the reduced model *mr* as follows and evaluated the significance of silencing by using the above method.

For the infusion experiment,

7 $${\mathsf{chose}}\_{\mathsf{right}} \sim {\mathsf{rl}}\_{\mathsf{delta}}\_{\mathsf{EV}}+({\mathsf{rl}}\_{\mathsf{delta}}\_{\mathsf{EV}}* {\mathsf{treatments}}\_{\mathsf{side}}| {\mathsf{subjid}})$$

For the optogenetic experiment,

8 $${\mathsf{chose}}\_{\mathsf{right}} \sim {\mathsf{rl}}\_{\mathsf{delta}}\_{\mathsf{EV}}+({\mathsf{rl}}\_{\mathsf{delta}}\_{\mathsf{EV}}* {\mathsf{treatments}}\_{\mathsf{side}}| {\mathsf{sessid}})$$

To estimate the shift in indifference point induced by bilateral FOF inactivation, we first fit a GLMM as described above. We generated synthetic data points for delta_EV to extend its range, and the model was used to predict *P*(Choose Lottery) for each synthetic data point. For each animal, we identified the delta_EV values that resulted in *P*(Choose Lottery) to be between 0.499 and 0.501, which is the definition of indifference point. The average indifference point was obtained by taking the mean of such values across animals.

To test whether unilateral PPC infusions led to an ipsilateral bias in both free choice and risk choice trials, we specified a GLMM as follows:

9 $${\mathsf{chose}}\_{\mathsf{ipsi}} \sim {\mathsf{infusion}}+({\mathsf{infusion}}| {\mathsf{subjid}})$$

where chose_ipsi is a binary variable indicating whether the animal chose the side ipsilateral to the infusion side or not, and infusion is a binary variable representing the presence of a unilateral PPC infusion.

To estimate changes in reaction time, we used linear mixed-effects models (LMMs). The formula for bilateral infusion full model (*mf*) was:

For the infusion experiment,

10 $$\log ({\mathsf{RT}}) \sim {\mathsf{treatments}}+{\mathsf{delta}}\_{\mathsf{EV}}* {\mathsf{choice}}+({\mathsf{delta}}\_{\mathsf{EV}}* {\mathsf{choice}}| {\mathsf{subjid}})$$

For the optogenetic experiment,

11 $$\log ({\mathsf{RT}}) \sim {\mathsf{treatments}}+{\mathsf{delta}}\_{\mathsf{EV}}* {\mathsf{choice}}+({\mathsf{delta}}\_{\mathsf{EV}}* {\mathsf{choice}}| {\mathsf{sessid}})$$

Whereas the reduced model (*mr*) was:

For the infusion experiment,

12 $$\log ({\mathsf{RT}}) \sim {\mathsf{delta}}\_{\mathsf{EV}}* {\mathsf{choice}}+({\mathsf{delta}}\_{\mathsf{EV}}* {\mathsf{choice}}| {\mathsf{subjid}})$$

For the optogenetic experiment,

13 $$\log ({\mathsf{RT}}) \sim {\mathsf{delta}}\_{\mathsf{EV}}* {\mathsf{choice}}+({\mathsf{delta}}\_{\mathsf{EV}}* {\mathsf{choice}}| {\mathsf{subjid}})$$

where $\log ({\mathsf{RT}})$ denotes the logarithm of reaction time, and choice is a binary value for the surebet/lottery choice (0/1). The LR test was performed using lrtest(mf, mr) to determine whether the treatment had a significant effect on the reaction time. Similarly, the formula for unilateral infusion full model (*mf*) was:

For the infusion experiment,

14 $$\log ({\mathsf{RT}}) \sim {\mathsf{treatments}}\_{\mathsf{sides}}+{\mathsf{rl}}\_{\mathsf{delta}}\_{\mathsf{EV}}* {\mathsf{choice}}+({\mathsf{rl}}\_{\mathsf{delta}}\_{\mathsf{EV}}* {\mathsf{choice}}| {\mathsf{subjid}})$$

For the optogenetic experiment,

15 $$\log ({\mathsf{RT}}) \sim {\mathsf{treatments}}\_{\mathsf{sides}}+{\mathsf{rl}}\_{\mathsf{delta}}\_{\mathsf{EV}}* {\mathsf{choice}}+({\mathsf{rl}}\_{\mathsf{delta}}\_{\mathsf{EV}}* {\mathsf{choice}}| {\mathsf{sessid}})$$

and the reduced model (*mr*) was:

For the infusion experiment,

16 $$\log ({\mathsf{RT}}) \sim {\mathsf{rl}}\_{\mathsf{delta}}\_{\mathsf{EV}}* {\mathsf{choice}}+({\mathsf{rl}}\_{\mathsf{delta}}\_{\mathsf{EV}}* {\mathsf{choice}}| {\mathsf{subjid}})$$

For the optogenetic experiment,

17 $$\log ({\mathsf{RT}}) \sim {\mathsf{rl}}\_{\mathsf{delta}}\_{\mathsf{EV}}* {\mathsf{choice}}+({\mathsf{rl}}\_{\mathsf{delta}}\_{\mathsf{EV}}* {\mathsf{choice}}| {\mathsf{sessid}})$$

To test whether the outcome of the previous trial affected choice on the current trial, we first classified the previous trial’s outcome into three categories: lottery-win, lottery-lose and surebet. If the previous trial was a violation, we considered that as a surebet choice (excluding post-violation trials did not change the results).

18 $${\mathsf{chose}}\_{\mathsf{lottery}} \sim {\mathsf{delta}}\_{\mathsf{ev}}+{\mathsf{prev}}\_{\mathsf{outcome}}\,+\left({\mathsf{delta}}\_{\mathsf{ev}}+{\mathsf{prev}}\_{\mathsf{outcome}}| {\mathsf{subjid}}\right)$$

where prev_outcome is a categorical variable with three levels of previous outcome as above.

To better check whether the perturbation had any effects on individual’s behavior performance, we did the LR test for each subject. We compared two models to test whether infusion and optogenetic silencing of FOF had an effect. The full model *mf* was:

19 $${\mathsf{chose}}\_{\mathsf{lottery}} \sim {\mathsf{delta}}\_{\mathsf{EV}}* {\mathsf{treatments}}$$

The reduced model *mr* was:

20 $${\mathsf{chose}}\_{\mathsf{lottery}} \sim {\mathsf{delta}}\_{\mathsf{EV}}$$

To probe whether the unilateral perturbation caused any left/right bias for each subject, the full model *mf* was:

21 $${\mathsf{chose}}\_{\mathsf{right}} \sim {\mathsf{rl}}\_{\mathsf{delta}}\_{\mathsf{EV}}* {\mathsf{treatments}}\_{\mathsf{side}}$$

The reduced model *mr* was:

22 $${\mathsf{chose}}\_{\mathsf{right}} \sim {\mathsf{rl}}\_{\mathsf{delta}}\_{\mathsf{EV}}$$

To test the changes in reaction time, for the bilateral perturbation, the full model *mf* was:

23 $$\log ({\mathsf{RT}}) \sim {\mathsf{treatments}}+{\mathsf{delta}}\_{\mathsf{EV}}* {\mathsf{choice}}$$

The reduced model *mr* was:

24 $$\log ({\mathsf{RT}}) \sim {\mathsf{delta}}\_{\mathsf{EV}}* {\mathsf{choice}}$$

For the unilateral perturbation, the full model *mf* was:

25 $$\log ({\mathsf{RT}}) \sim {\mathsf{treatments}}\_{\mathsf{sides}}+{\mathsf{rl}}\_{\mathsf{delta}}\_{\mathsf{EV}}* {\mathsf{choice}}$$

The reduced model *mr* was:

26 $$\log ({\mathsf{RT}}) \sim {\mathsf{rl}}\_{\mathsf{delta}}\_{\mathsf{EV}}* {\mathsf{choice}}$$

The variable name had the same meanings as the above formulas. The LR test was performed using lrtest(mf, mr) to determine whether the treatment had a significant effect on the animals’ performance.

#### Surebet learning

To test the role of PPC in learning, we periodically changed the surebet magnitude in a model-based way to shift the decision boundary. For each shift, we fit the three-agent model (described below) on control data from the past 14 d to obtain a set of parameters. Using a binary search algorithm, we then used those parameters to generate synthetic choices with different surebet magnitudes until we found a value that produced a shift in probability choosing lottery (*P*(Choose Lottery)) close to the target (drawn uniformly from ±*U*(0.2, 0.3)). The new surebet magnitude was assigned to the animal on the day of change. All animals in the surebet learning experiment had undergone two rounds of shift without any infusion, in the course of 14 d, to acclimate them to the new routine before bilateral PPC infusions. The first two surebet change sessions are not included in the analysis of Fig. 6.

To test whether bilateral PPC infusions (0.6 mg kg^−1^) changed the slope of the actual shift in response to a predicted shift, we fit a linear model *mf*

27 $${\mathsf{actual}}\_{\mathsf{shift}} \sim {\mathsf{predicted}}\_{\mathsf{shift}}+{\mathsf{predicted}}\_{\mathsf{shift}}:{\mathsf{infusion}}$$

To check the whether infusion change the slope, we drop the interaction term *predicted*_*shift* : *infusion* from the above model and fit a reduced model *mr* as follows:

28 $${\mathsf{actual}}\_{\mathsf{shift}} \sim {\mathsf{1}}+{\mathsf{predicted}}\_{\mathsf{shift}}$$

The LR test was performed using lrtest(mf, mr) to determine whether the treatment had a significant effect on the slope.

#### The three-agent mixture model

We developed a three-agent mixture model that used four parameters to transform the offers on each trial into a probability of choosing lottery as a weighted outcome of three agents (Fig. 3a): a rational agent, a ‘lottery’ agent and a ‘surebet’ agent. For the rational agent, we assume an exponential term *ρ* for the utility function, *U* = *V*^*ρ*^. A concave utility function (*ρ* < 1) implies risk aversion; a linear function with *ρ* = 1 implies being risk neutral; and a convex function (*ρ* > 1) implies risk seeking. We captured stochasticity in the animals behavior by modeling the internal representation of expected utility as a Gaussian random variable.

29 $$E{U}_{L} \sim {{{\mathcal{N}}}}\left({V}_{L}^{\,\rho }{P}_{L},\sigma \right)$$

30 $${U}_{SB} \sim {{{\mathcal{N}}}}\left({V}_{SB}^{\,\rho },\sigma \right)$$

where the expected utility of lottery, *EU*_*L*_, and the utility of the surebet, *U*_*SB*_, are Normal distributions. *V*_*L*_, *V*_*SB*_ refer to the magnitude of lottery and surebet, and *P*_*L*_ is the probability of lottery payout. The probability of choosing lottery for the rational agent then becomes

31 $${p}_{{\mathsf{Choose}}\,{\mathsf{Lottery}}}^{\,rational}=p\left(E{U}_{L} > {U}_{SB}\right)$$

32 $$=p\left(E{U}_{L}-{U}_{SB} > 0\right)$$

33 $$=p\left({{{\mathcal{N}}}}\left({V}_{L}^{\,\rho }{P}_{L},\sigma \right)-{{{\mathcal{N}}}}\left({V}_{SB}^{\,\rho },\sigma \right) > 0\right)$$

34 $$=p\left({{{\mathcal{N}}}}\left({V}_{L}^{\,\rho }{P}_{L}-{V}_{SB}^{\,\rho },\sqrt{2}\sigma \right) > 0\right)$$

35 $$=1-{{\Phi }}\left(0;{V}_{L}^{\,\rho }{P}_{L}-{V}_{SB}^{\,\rho },\sqrt{2}\sigma \right)$$

where ${{\Phi }}(0;{V}_{L}^{\,\rho }{P}_{L}-{V}_{SB}^{\,\rho },\sqrt{2}\sigma )$ is the cumulative Normal distribution with mean ${V}_{L}^{\,\rho }{P}_{L}-{V}_{SB}^{\,\rho }$, standard deviation $\sqrt{2}\sigma$ and evaluated at 0. Note that this provides fits with similar likelihood as the softmax choice function with *β* as temperature:

36 $${{\Delta }}EU={V}_{L}^{\,\rho }{P}_{L}-{V}_{SB}^{\,\rho }$$

37 $$p({\mathsf{Choose}}\,{\mathsf{Lottery}})=\frac{1}{1+{e}^{-\beta {{\Delta }}EU}}$$

The other two agents in the three-agent mixture model are the lottery and surebet agents. They represent the habitual bias of the animal to make one or the other choice regardless of the lottery offer, similar to biased lapse terms in ref. ^19^. The probability of choosing lottery for the lottery agent is ${p}_{{\mathsf{Choose\,Lottery}}}^{lottery}=1$ and for the surebet agent is ${p}_{{\mathsf{Choose\,Lottery}}}^{surebet}=0$.

The last step is to obtain *P*(Choose Lottery) by mixing the probability from each agent $\overrightarrow{P}$ with their respective mixing weights $\overrightarrow{\omega }$ that sum up to 1. Formally,

38 $$P({\mathsf{Choose}}\,{\mathsf{Lottery}})=\bf{P}\cdot \bf{\upomega }$$

39 $${={P}_{\mathsf{Choose}\,\mathsf{Lottery}}^{rational}}{{\omega }_{rational}}+1 {{\omega}_{lottery}}+0 {{\omega}_{surebet}}$$

40 $$\sum \overrightarrow{\omega }=1$$

#### Model fitting

We estimated the posterior distribution over model parameters with weakly informative priors using the rstan package (version 2.21.2, Stan Development Team, 2020). rstan is the R interface of Stan, a probabilistic programming language that implements a Hamiltonian Monte Carlo algorithm for Bayesian inference. Six Markov chains with 13,000 samples each were obtained for each model parameter after 8,000 warm-up samples. The $\hat{R}$ convergence diagnostic for each parameter was close to 1, indicating that the chains mixed well.

To improve model convergence, we use ‘raw’ parameters that were transformed into the variables described in the equations above as follows:

41 $$\rho ={e}^{\phi }$$

42 $$\sigma ={e}^{\psi }$$

43 $${\omega }_{rational}=logistic({\omega }_{1})$$

44 $${\omega }_{lottery}=\left(1-{\omega }_{rational}\right)logistic({\omega }_{2})$$

45 $${\omega }_{surebet}=\left(1-{\omega }_{rational}\right)\left(1-logistic\left({\omega }_{2}\right)\right)$$

The model’s raw parameters included *ϕ*, with a prior of ${{{\mathcal{N}}}}(0,0.5)$; *ψ*, with a prior of ${{{\mathcal{N}}}}(-3,0.3)$; *ω*_1_, with a prior of ${{{\mathcal{N}}}}(3,1)$, equivalent to *ω*_*rational*_ after a *logistic* transformation; and *ω*_2_ with a prior of ${{{\mathcal{N}}}}(0,1)$, representing the proportion of the surebet agent in 1 − *logistic*(*ω*_1_) after the *logistic* transformation, where *logistic*(*x*) = 1/(1 + *e*^−*x*^). We refer to these four parameters as *control* parameters, because they capture the behavior on control trials.

This parameterization allowed us to treat the effects of perturbations as shifts of the raw parameters while guaranteeing that transformed parameters were constrained (for example, *ρ* > 0, *σ* > 0, ∑*ω* = 1).

For each inactivation dataset, we added a new parameter for each raw parameter to estimate the effects of inactivation:

46 $$\rho ={e}^{\phi +{{\Delta }}\phi }$$

47 $$\sigma ={e}^{\psi +{{\Delta }}\psi }$$

48 $${\omega }_{rational}=logistic({\omega }_{1}+{{\Delta }}{\omega }_{1})$$

49 $${\omega }_{lottery}=\left(1-{\omega }_{rational}\right)logistic({\omega }_{2}+{{\Delta }}{\omega }_{2})$$

50 $${\omega }_{surebet}=\left(1-{\omega }_{rational}\right)\left(1-logistic({\omega }_{2}+{{\Delta }}{\omega }_{2})\right)$$

Where Δ*ϕ* denotes the change in *ρ* in the log space, it had a prior of ${{{\mathcal{N}}}}(0,0.5)$; Δ*ψ*, with a prior of ${{{\mathcal{N}}}}(0,0.5)$, represents how the infusions could shift noise; and Δ*ω*_1_ (${{{\mathcal{N}}}}(0,1)$) and Δ*ω*_2_ (${{{\mathcal{N}}}}(0,1)$) fit potential changes in *ω*_1_ and *ω*_2_ before the *logistic* transformation, respectively. We refer to these four parameters as Δ parameters, because they capture the change due to perturbation.

In addition to the base and Δ parameters described above, each subject could deviate from the base parameters, and the priors for how much the subjects could deviate from the base parameters were as follows: Δ*ϕ*${{{\mathcal{N}}}}(0,0.35)$, Δ*ψ*${{{\mathcal{N}}}}(0,0.35)$, Δ*ω*_1_${{{\mathcal{N}}}}(0,0.35)$, Δ*ω*_2_${{{\mathcal{N}}}}(0,0.35)$. The process of selecting the priors involved sampling from the priors of the hierarchical model and inspecting the samples for long tails (which would often result in divergent transitions in the synthetic fits) and fitting the synthetic data to check for divergent transitions and accuracy in recovering the generative parameters. We implemented the models using the brms (version 2.17.0) R package^29^, a wrapper for Stan.

#### Synthetic datasets

**Extended Data Fig. 5:**
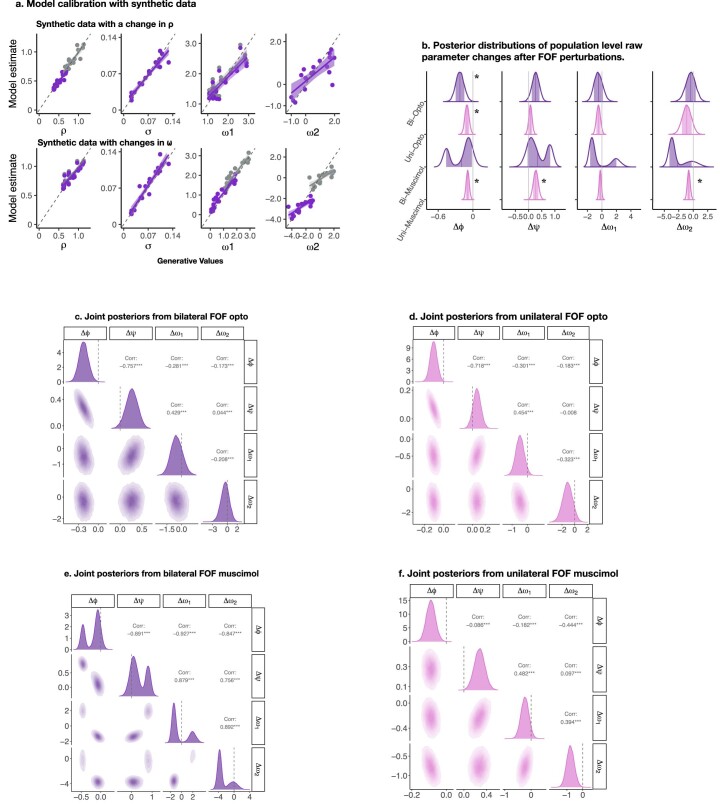
Model diagnostics and details of FOF fits. **a**. 3-agent model fits to synthetic data where we simulated Δ*ρ* = − 0.5 (top row) or Δ*ω*_1_ = − 1 and Δ*ω*_2_ = − 3. The 3-agent model correctly captures control (grey) and perturbed (purple) parameters (n = 20 simulated subjects, the dark line and error band represent the linear fit of the parameters and 95% confidence intervals). **b**. The posterior distributions of the raw model parameters (see Methods for definitions of *ϕ*, *ψ*, *ω*_1_, *ω*_2_, A star (*) indicates that 97.5% of the posterior was not overlapping with 0. Bi-Opto: n = 5 rats, Uni-Opto: n = 8 rats, Bi-Muscimol: n = 8 rats, Uni-Muscimol: n = 8 rats). **c-f**. Two-dimensional joint posteriors of the *perturbation* parameters (Bi-Opto: n = 5 rats, Uni-Opto: n = 8 rats, Bi-Muscimol: n = 8 rats, Uni-Muscimol: n = 8 rats). **c,d**. Although, there is a small trade-off between changes in parameters, the tight overall distribution of Δ*ϕ* indicates a high degree of confidence of a change in *ρ* (Eq. (46)). **e**. The posteriors for the bilateral muscimol experiment suggest that there are two possible explanations for the data. Either there was a shift in *ρ* or there was a shift in the mixing fraction *ω*. **f**. As in **d** but for muscimol silencing.

To test the validity of our model, we created synthetic datasets with parameters that generated psychometric curves qualitatively similar to our data and generated perturbations that were either changes in *ρ* or changes in *ω*. The three-agent model was fit to the synthetic datasets, and it was able to recover the generative parameters accurately (Extended Data Fig. 5a).

### Dynamical models

We generated a six-node rate model as a potential mechanism for understanding how muscimol inactivation of the FOF could cause a reduction in lottery choices via a change in the curvature of the utility function. The activity of the six nodes, *X*, is governed by the following equations, where *v* is the magnitude of the lottery, and the *i* in *g*(*v*, *t*, *i*) represents the node index (1–6). Simulation was done using Euler’s method in Julia^66^:

51 $$dX=dt(-X/\tau +WX+g(v,t,i)+{{{\mathcal{N}}}}(0,\sigma ))$$

52 $$X=f(X+dX\,)$$

53 $$f(x)=\left\{\begin{array}{ll}x&\,{{\mbox{if}}}\,0\le x\le 100\\ 0&\,{{\mbox{if}}}\,x < 0\\ 100&\,{{\mbox{if}}}\,x > 100\end{array}\right.$$

54 $$g(v,t,i)=\left\{\begin{array}{ll}0&\,{{\mbox{if}}}\,0.1 > t > 1\,{{{\rm{s}}}}\,or\,i\le 3\\ v\tau &\end{array}\right.$$

55 $$\tau =0.15\,{{{\rm{s}}}}$$

56 $$dt=0.001\,{{{\rm{s}}}}$$

57 $$\sigma =0.3{dt}^{-1}$$

58 $$for\,{w}_{ij}\in W,$$

59 $${w}_{ij} \sim {{{\mathcal{N}}}}(5/6,0)$$

We began the simulation of each trial a few seconds before the input was turned on to allow the network to reach its baseline fixed point. We examined different instantiations of this model by generating the weight matrix, *W*, from different random seeds. Many (but not all) of these networks gave qualitatively similar results. The seed used to generate *W* for the plots in Fig. 4 paper was 131.

**Extended Data Fig. 6:**
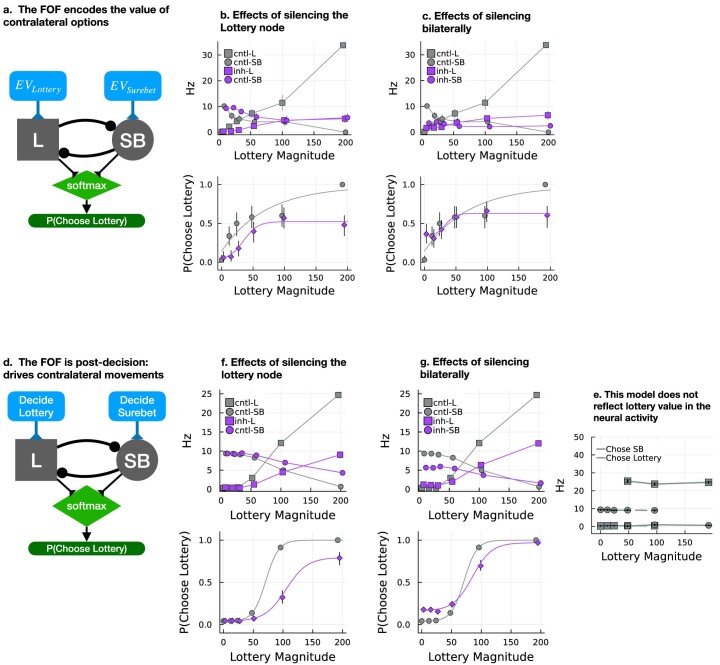
Alternative dynamical models. We tested whether two alternative models of FOF function could explain our findings. In both models, we refer to the the FOF contralateral to the lottery as the lottery node, *L* (square), and the FOF contralateral to the surebet as the surebet node, *SB* (round). These shapes also correspond to the neural activity plots in the upper panels of b,c,f,g. **a**. The input into each node of the FOF is the expected value (*EV*) of the corresponding offer. As such, the neurons in this model correlate with lottery magnitude. **b**. Upper panel: unilateral silencing of *L* dramatically decreases the firing rate of *L* (compare the grey and purple squares). The dots represent the mean network responses across 20 trials (per lottery, *n* = 6 × 20 = 120 simulated trials). The error bars represent the 95% confidence interval of the mean across 200 permutations. Lower panel: Silencing *L* results in a dramatic behavioral shift away from choosing the lottery - a contralateral impairment. Silencing *SB* would also result in a contralateral impairment (inconsistent with our findings). The dots represent the mean P(choose lottery) based on the activity shown in the upper panel. The error bar is the 95% CI of the mean. The error bars represent the 95% confidence interval of the mean across 200 permutations. **c**. as in b for bilateral silencing. Here, the behavioral effect is an increase in noise, not a shift away from the lottery. **d**. In this model there is an upstream process that decides whether to choose the lottery or surebet, and the FOF gets as input this binary decision^25^. **f,g** as in b,c. The unilateral effects are large and bilateral silencing increases noise. **e**. Since the input the FOF in this model is post-decision, the neurons in this model do not correlate with lottery magnitude after conditioning on choice.

**Extended Data Fig. 7:**
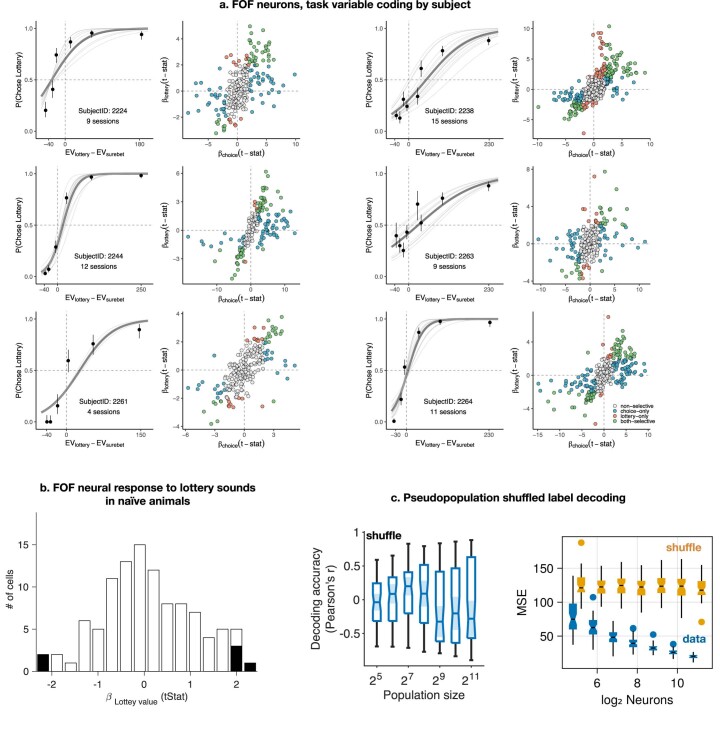
Electrophysiological data and controls. **a**. Single-neuron task coding by subject. For each subject, the left panel shows the rat’s choice behavior for all the electrophysiology recording sessions. The dots with error bars show the probability of choosing lottery against ΔEV of the two options. The lines are the psychometric curves estimated by a logistic fit to the data, the thin gray lines are fit to each session, the thick gray line fit to all the sessions combined. The right panel shows the distribution of the *t*-statistic for lottery value (y-axis) and upcoming choice (x-axis) for all the neurons recorded in each animal. Gray dots indicate the non-task relevant neurons, light blue dots indicate the pure choice neurons, orange dots indicate the pure lottery selectivity neurons, and green dots indicate tuning for both upcoming choice and lottery values (n = 893 trials, 9 sessions for subject 2224; n = 1,754 trials, 15 sessions for subject 2238; n = 1,421 trials, 12 sessions for subject 2244; n = 1,228 trials, 9 sessions for subject 2263; n = 430 trials, 4 sessions for subject 2261; n = 1,040 trials, 11 sessions for subject 2264, the circles with error bars are the mean and 95% binomial confidence intervals.). **b**. We recorded neurons from the FOF of 4 rats to test whether the relationship between firing rate and lottery could be due to FOF encoding the percept of the different lottery sounds. Out of the 105 neurons recorded, only 6 had *p* < 0.05 encoding of lottery cues, which was not significantly different than expected by chance (*χ*^2^(1, 105) = 0.051, *p* = 0.82, one-sided). **c**. Left: Decoding accuracy (Pearson’s *r*) for pseudopopulation decoding with shuffled training labels. With only 6 lotteries, the correlation can be very high by chance, but the distributions of accuracy are clearly distinct from the real decoding. Right: Comparing decoding using mean squared error (MSE) instead of *r*. Using MSE avoids the problems of computing correlation with small *n*. The decoding with the real data is significantly better than the shuffled data for all population sizes (n = 50 pseudosessions, all *p* < 0.00001, The box whisker plots show the median, lower/upper quartile, minimum/maximum and the outliers of the data, the notch showed the $median\pm (1.57\times interquartilerange)/\sqrt{n}$, not adjusting for multiple comparisons).

**Extended Data Fig. 8:**
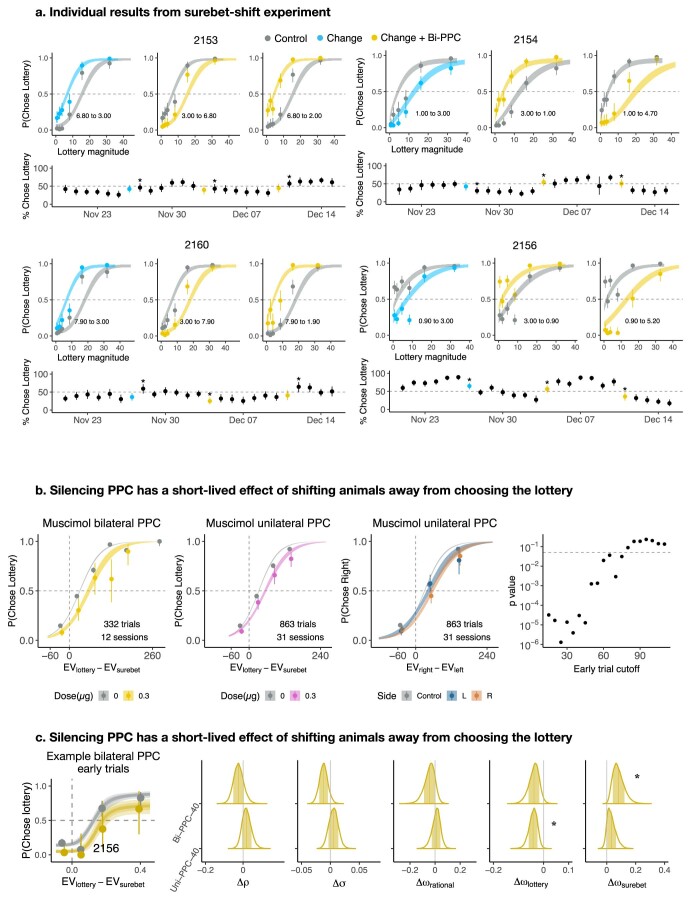
Role of the PPC in re-categorisation and the risky-choice task. **a**. Behavioral adaptation of subjects 2153, 2154, 2156 and 2160 in the surebet learning experiment. For each animal, we fit a model to the control trials and used it for predicting the shifts. *Top three subpanels*: the circles with error bars are the binned mean and 95% binomial confidence intervals; the ribbons are generated using the fit parameter posterior of with 80% confidence intervals. behavior from 6 sessions immediately before a surebet change is in gray, behavior from 7 sessions after a surebet change (including the very day) is in light blue if no infusion, in gold if with 0.6 *μ*g bilateral PPC infusion. Text annotation shows the old and new surebet magnitudes. *Bottom subpanel*: The chose lottery % of each session. Asterisk indicates when change in choices can be significantly detected on that session compared to the previous 6 sessions with old surebet magnitude. **b**. The psychometric plots are versions of Fig. 2b,e,h but using only the first 40 trials in each session. The rightmost plot show the *p*-value of the infusion from GLMM model as a function of the the cut-off for including trials as ‘early’. The behavioral effect of PPC silencing on risky choice lasted only about 45 trials. Including more then 45 trials begins to wash out the effect and after 80 trials there is no longer a significant effect (n = 7 rats, the circles with error bars are the mean and 95% binomial confidence intervals, z-test, two-sided, not adjusting for multiple comparisons.). **c**. 3-agent fits to the fits 40 trials of PPC muscimol sessions. The model suggests that the effect of PPC silencing is on the bias parameters, particular a decrease in *ω*_*lottery*_ and increase in *ω*_*surebet*_. This can be seen in the psychometric plot from 2156 (left panel, n = 40 trials, the circles with error bars are the binned mean and 95% binomial confidence intervals, the dark and light shade represent model predicted 50%, 80% confidence intervals.). The change due to PPC silencing appears to be a downward shift (n = 7 rats, a star (*) indicates that 97.5% of the posterior was not overlapping with 0).

We additionally generated two two-node models where one node represented the FOF contralateral to the lottery, *L*, and the other the FOF contralateral to the surebet, *SB* (Extended Data Fig. 6). For the first two-node model, the parameters were the same as above, except:

60 $$\sigma =0.1{dt}^{-1}$$

61 $$W=\left(\begin{array}{l}0\quad\ -4\\ -4\quad\ 0\end{array}\right)$$

62 $$g(v,t,i)=\left\{\begin{array}{ll}0&\,{{\mbox{if}}}\,0.1 > t > 1\,{{{\rm{s}}}}\\ E{V}_{lottery}\tau &\,{{\mbox{if}}}\,i=L\\ E{V}_{surebet}\tau &\,{{\mbox{if}}}\,i=SB\end{array}\right.$$

The second two-node model was the same as the first, except that its input was post-decision. On each trial of the simulation, an upstream process emulated a rational agent (*ρ* = 0.7) in choosing between the surebet and the lottery using a softmax decision rule. We denote trials where the upstream process chose the lottery as *C*_*L*_ and trials where the agent chose the surebet as *C*_*SB*_.

63 $$g(v,t,i)=\left\{\begin{array}{ll}0&\,{{\mbox{if}}}\,0.1 > t > 1\,{{{\rm{s}}}}\\ 0&\,{{\mbox{if}}}\,(i=L)\wedge {C}_{SB}\\ 0&\,{{\mbox{if}}}\,(i=SB)\wedge {C}_{L}\\ 100\tau &\,{{\mbox{if}}}\,(i=L)\wedge {C}_{L}\\ 100\tau &\,{{\mbox{if}}}\,(i=SB)\wedge {C}_{SB}\end{array}\right.$$

### Electrophysiology

After the surgery, the rats recovered for 6 d with ad libitum access to food and water. Then, the rats were returned to water restriction and resumed behavior and electrophysiology recording on the seventh postoperative day. Neural activity was digitized at 30 kHz, amplified and bandpass filtered at 0.6–7,500 Hz using a 64-channel Intan headstage (C3325, Intan Technologies); the SPI cable of the Intan headstage (C3203, Intan Technologies) was tethered to a commutator (MMC250, Shenzhen Moflon Technology); and all the raw data were processed using an Open Ephys acquisition board (https://open-ephys.github.io/acq-board-docs/) connected to a computer to visualize and store the neural signals.

During the recording, at the end of each trial, a serial TTL message encoding the current trial number was sent from our behavioral control hardware to the acquisition system to synchronize the neural signal with the behavioral data. The probes were turned down ~ 100 μm every 4–6 d until the white matter was reached.

Offline spike sorting was performed by using Kilosort version 2 with the default setting. Spike clusters were manually curated using Phy. The quality metrics and waveform metrics for sorted units were computed using ecephys spike sorting (https://github.com/AllenInstitute/ecephys_spike_sorting). Specifically, we selected units with an average firing rate >1 Hz, a signal-to-noise ratio >1.5 and a presence ratio >0.95 over the course of recording sessions.

The four naive rats (used to examine the sensory response of the FOF to the stimuli) were not water restricted. Recordings took place in the same behavioral chambers, and FOF neural activity was recorded while rats passively listened to six distinct lottery cues. Approximately 300 lottery cues were played for each passive listening session. The other details of recording were the same for these animals.

#### Single-neuron analyses

For the example neuron raster and peri-stimulus time histogram (PSTH) plots, spike times were aligned with the sound cue in a 1.2-s time window (−0.1 s before the cue onset and 0.1 s after the fixation end) with the bin size set to 10-ms resolution and smoothed with a causal half Gaussian kernel (standard deviation of 20 ms).

To determine whether the firing of the cell could predict the upcoming choice selection during the fixation period, we counted the spikes on each trial in the late fixation period (0.5–1 s after the cue onset). We then ran a mixed-effects linear regression to see how choice affected neural responses in each time window. Single-cell mixed-effects linear models were fit in MATLAB using *fitlme* with the following formula:

64 $${\sf{zscore}}\_{\sf{spike}}\_{\sf{counts}} \sim {\sf{chose}}\_{\sf{lottery}}+({{1}}| {\sf{lottery}}\_{\sf{magnitude}})$$

where ${\sf{zscore}}\_{\sf{spike}}\_{\sf{counts}}$ was *z*-scored spike counts for that time window; chose_lottery was a binary value, set to 1 if the lottery was chosen on that trial, and otherwise it was 0. lottery_magnitude was six relative reward values (0.5, 2, 4, 8, 16 and 32) corresponding to the six distinct sound cues. *P* < 0.05 for the coefficient of the fixed parameter chose_lottery was used to identify a choice selective cell. By putting lottery_magnitude as a random effect, we can ensure that a significant coefficient for chose_lottery is not due to a spurious influence of lottery magnitude on choice-related activity.

Another mixed-effects linear regression was implemented to evaluate the contribution of different lottery magnitudes to spike firing in FOF regardless of choice. This was fit using the MATLAB function *fitlme* with the following formula:

65 $${\sf{zscore}}\_{\sf{spike}}\_{\sf{counts}} \sim {\sf{lottery}}\_{\sf{magnitude}}+\left({{1}}| {\sf{chose}}\_{\sf{lottery}}\right)$$

*P* < 0.05 for the coefficient of the fixed parameter lottery_magnitude was used to identify a lottery tuning cell. By putting chose_lottery as a random effect, we can ensure that a significant coefficient for lottery_magnitude is not due to a spurious influence of choice on magnitude-related activity.

We validated the results from the mixed-effects linear models with non-parametric permutation shuffling methods as follows. We randomly permuted the firing rates across trials and then refit the models to estimate the coefficients for lottery magnitude and choice. We performed this randomization 10,000 times and considered a cell to be significant if the *β* value from the data was outside the 95% CI of the shuffled *β* distribution. This non-parametric procedure gave close results to the original.

#### Pseudopopulation decoding

To generate a single pseudosession, we sampled *N* cells (for *N* ∈ 2^5:11^) with replacement. We also ran the analyses sampling without replacement and obtained similar results. For each selected cell, we excluded the trials where the subject chose the surebet. Note that we also checked lottery decoding from surebet-only trials and found a similar result. We split the trials for each lottery magnitude for each cell in half (into test and training sets). Then, we resampled within each set so that there were 20 training trials and 20 test trials for each of the six lottery magnitudes for each cell. Thus, we generated a 120 × *N* matrix of *z*-scored spike counts during the 500-ms window before the go-cue (‘late fixation’) for training, *X*, and another of the same size for testing, *W*. We then performed principal component analysis on the training matrix, *X*; took the top four principal components; and projected our data to get 120 × 4 training, *X*_*r*_, and testing, *W*_*r*_, matrices. Then, we used linear regression to estimate coefficients, *B*, such that *L* = *X*_*r*_*B* + *ϵ*, where *L* is the true lottery magnitude, and $\hat{L}={W}_{r}B$ was computed from the test data. Finally, we computed the cross-validated mean squared error (MSE) and Pearson correlation, *r*, between the true lottery magnitude, *L*, and the estimate $\hat{L}$. Due to our procedure (of sampling 20 trials of each magnitude), *L* had the true labels for both *X*_*r*_ and *W*_*r*_. We generated 50 pseudosessions for each *N*. To show that our decoding was above chance levels, we repeated the procedure, shuffling the labels, *L*. The code for this procedure was written in Julia using Pluto.jl (ref. ^67^), and then results were imported into MATLAB for plotting, to preserve visual consistency with the other panels in the figure.

#### Single-trial decoding

To decode each session, we excluded forced and violation trials and created a *T* × *N* matrix, *Z*_*raw*_, of *z*-scored (by cell) spike counts in the 500-ms ‘late fixation’ window, where *T* is the number of included trials, and *N* is the number of neurons in the session. We also had two corresponding length *T* vectors: **C**, which was 1 for chose-lottery trials and 0 for chose-surebet trials, and *L*, which was the normalized lottery magnitude on each trial (*L* = *L*_*raw*_ / *max*(*L*_*raw*_)). Then, we performed principal component analysis on *Z*_*raw*_, took the top four principal components and projected the data to get a *T* × 4 matrix, *Z*. We created an index, *I* = [*i* for *i* ∈ 1. . *T* if *C*_*i*_ = 1], of the trials where the subject chose the lottery and then shuffled the index, *I*_*s*_ = *shuffle*(*I*). We then performed a 20-fold cross-validation such that on, for example, the third fold, the third 5% of trials in *I*_*s*_ were designated as test trials and the rest as training trials. Let *g* designate the trials in the training set and *h* indicate the trials in the test set. We fit a linear model, *L*_*g*_ = *Z*_*g*_*B*_*g*_ + *ϵ*, and then generated a cross-validated prediction ${\hat{L}}_{h}={Z}_{h}{B}_{g}$. After going through all the folds, we fit a model, *L*_*I*_ = *Z*_*I*_*B*_*I*_ + *ϵ*, on all trials where **C** = 1 and used the coefficient *B*_*I*_ to estimate the lottery magnitude for trials where *C* = 0 (surebet trials). Thus, at the end of the procedure, we had a length *T* vector, $\hat{{\bf {L}}}$, of cross-validated estimates of the lottery magnitude on each trial, and we computed the Pearson correlation $r={\mathrm{cor}}\,(L,{\hat{L}})$ as a measure of decoding accuracy for that session.

For both population decoding methods, we noted that large lotteries were underestimated. We fit two parameters, *α*, *ρ*, using a power law model $\hat{{\bf {L}}}$ = *f*(*L*) = *αL*^*ρ*^, where *L* is the normalized original lottery magnitudes, and $\hat{L}$ is the linear model estimated lottery magnitudes. Then, we computed the correlation between $\hat{{\bf {L}}}$ and *f*(*L*), *r*_*nl*_ = cor(*f*(*L*), $\hat{{\bf {L}}}$). We used *r*_*nl*_ as a measure of decoding accuracy. We also computed the MSE, $MSE=\frac{1}{n}\sum_{i=1}^n {(L_i-{\hat{L}}_i)}^{2}$, because correlation can give high values with only six distinct lottery magnitudes, even for shuffled data.

#### Analysis of FOF responses during passive listening

To test whether the firing rate of FOF neurons is correlated with the lottery cues, FOF neural responses to lottery cues were recorded from four animals while they passively listened to the cues. These four animals were never trained for the risky choice task. We counted the spikes using the same time window as for the behavioral tasks (0.5–1 s after the cue onset). We then performed linear regression to see whether the FOF neural responses in each time window was correlated with the physical property of the lottery cue. Single-cell linear models were fit in MATLAB using *fitlm* with the following formula:

66 $${\sf{zscore}}\_{\sf{spike}}\_{\sf{counts}} \sim {\sf{lottery}}\_{\sf{cue}}$$

Where ${\mathsf{zscore}}\_{\mathsf{spike}}\_{\mathsf{counts}}$ was *z*-scored spike counts for that time window. lottery_cue was six lottery sounds, which is the same as the sound cues used for the risky behavior task. *P* < 0.05 for the coefficient of lottey_cue was used to identify a lottey_cue selectivity cell. The χ^2^ test was performed to check whether the number of lottey_cue selectivity cells was significantly different from chance level.

### Reporting summary

Further information on research design is available in the Nature Portfolio Reporting Summary linked to this article.

## Online content

Any methods, additional references, Nature Portfolio reporting summaries, source data, extended data, supplementary information, acknowledgements, peer review information; details of author contributions and competing interests; and statements of data and code availability are available at 10.1038/s41593-023-01461-x.

### Supplementary information

Supplementary Information Supplementary Figs. 1–4, Tables 1–5 and Statistical Appendices 1–9.

Reporting Summary

## Data Availability

Data are available at https://github.com/erlichlab/risk-fof-ppc-2023.

## Code availability

Code is available at https://github.com/erlichlab/risk-fof-ppc-2023.
