# Supplementary material for: The rat frontal orienting field dynamically encodes value for economic decisions under risk
Source: Nat Neurosci. 2023 Oct 19;26(11):1942–52. doi: 10.1038/s41593-023-01461-x (PMC10620098; doi:10.1038/s41593-023-01461-x)
Supplement: Supplementary file 1 — Supplementary Figs. 1–4, Tables 1–5 and Statistical Appendices 1–9. [file 41593_2023_1461_MOESM1_ESM.pdf]

nature neuroscience

Article https://doi.org/10.1038/s41593-023-01461-x

The rat frontal orienting field dynamically encodes value for economic decisions under risk

In the format provided by the authors and unedited

The rat frontal orienting field dynamically encodes value for economic decisions under risk.

Supplementary Information

Chaofei Bao, Xiaoyue Zhu, Joshua Moller-Mara, Jingjie Li, Sylvain Dubroqua, Jeffrey C. Erlich

Contents

1 Supplementary Figures **3**
1.1 Soft-fixation behavior . . . 3
1.2 Timeline of muscimol experiments . . . 4
1.3 Histology . . . 5
1.4 Influence of silencing FOF and PPC on Reaction times . . . 6

2 Supplementary Tables **7**
2.1 Bilateral opto fits . . . 7
2.2 Unilateral opto fits . . . 7
2.3 Muscimol fits . . . 8
2.4 Electrophysiology fits . . . 9
2.5 Experimental Parameters . . . 10

3 Statistical Appendix **11**
3.1 Figure 2b: Muscimol bilateral PPC . . . 11
3.1.1 Model Summary . . . 11
3.1.2 Likelihood Ratio Test . . . 11
3.2 Figure 2c: Muscimol bilateral FOF . . . 12
3.2.1 Model Summary . . . 12
3.2.2 Likelihood Ratio Test . . . 12
3.3 Figure 2d: Opto bilateral FOF . . . 13
3.3.1 Model Summary . . . 13
3.3.2 Likelihood Ratio Test . . . 13
3.4 Figure 2e: Muscimol unilateral PPC . . . 14
3.4.1 Model Summary . . . 14
3.4.2 Likelihood Ratio Test . . . 14
3.5 Figure 2f: Muscimol unilateral FOF . . . 15
3.5.1 Model Summary . . . 15
3.5.2 Likelihood Ratio Test . . . 15
3.6 Figure 2g: Opto unilateral FOF . . . 16
3.6.1 Model Summary . . . 16
3.6.2 Likelihood Ratio Test . . . 16
3.7 Figure 2h: Muscimol unilateral PPC (left vs. right) . . . 17
3.7.1 Model Summary . . . 17
3.7.2 Likelihood Ratio Test . . . 17
3.8 Figure 2i: Muscimol unilateral FOF (left vs. right) . . . 18
3.8.1 Model Summary . . . 18
3.8.2 Likelihood Ratio Test . . . 18
3.9 Figure 2j: Opto unilateral FOF (left vs. right) . . . 19
3.9.1 Model Summary . . . 19
3.9.2 Likelihood Ratio Test . . . 19

1 Supplementary Figures

1.1 Soft-fixation behavior

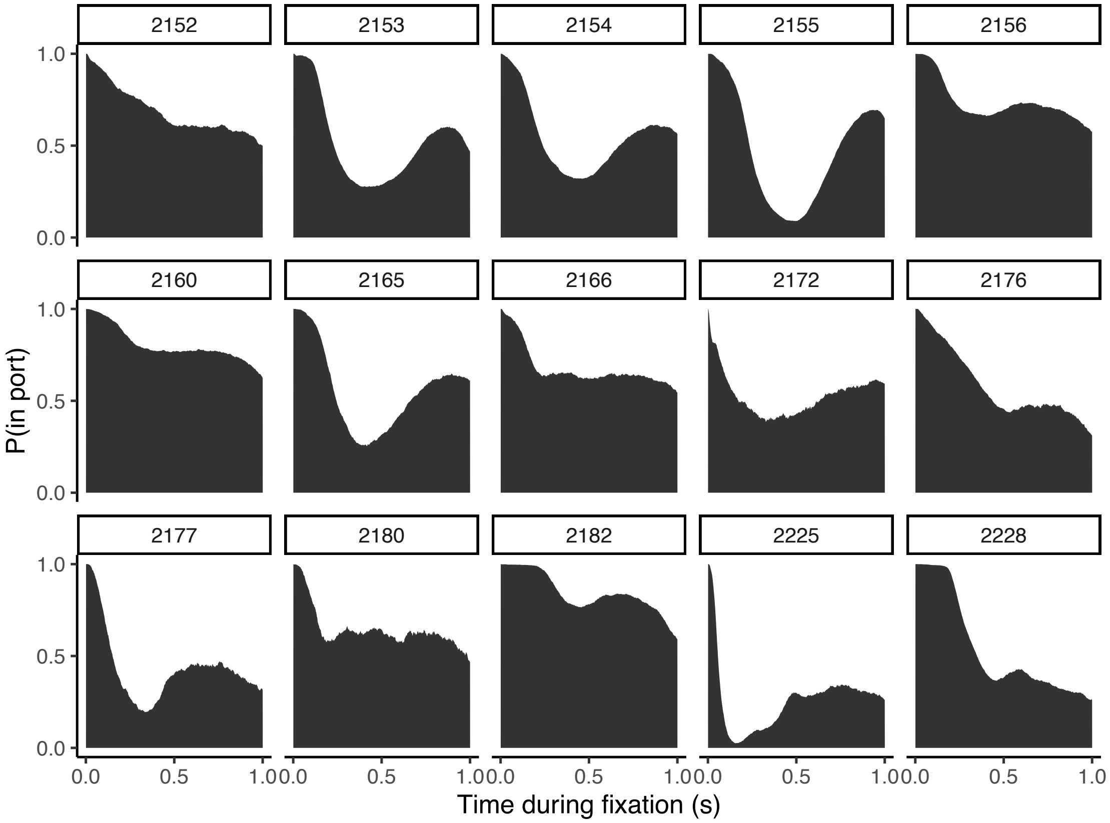

Figure 1. 'Soft-fixation' behaviour. The probability of being in the center port between the first center port and the go-cue. For each trial, the period between the initial poke and the go-cue was segmented into 1000 bins. Go-cue is defined as the onset of the choice port lights. For each bin, a binary value was obtained to indicate whether the animal was in the port or not. The probability of being in the port for each bin was calculated by taking the mean of the binary vector. Only control trials were used for this analysis. Note: all the electrophysiology data and most optogenetic data was collected from sessions where rats were required to remain in the start port for the entire fixation period (n = 15 rats). On the vast majority of trials, animals did not leave the start port for more than 200ms during the soft-fixation window

1.2 Timeline of muscimol experiments

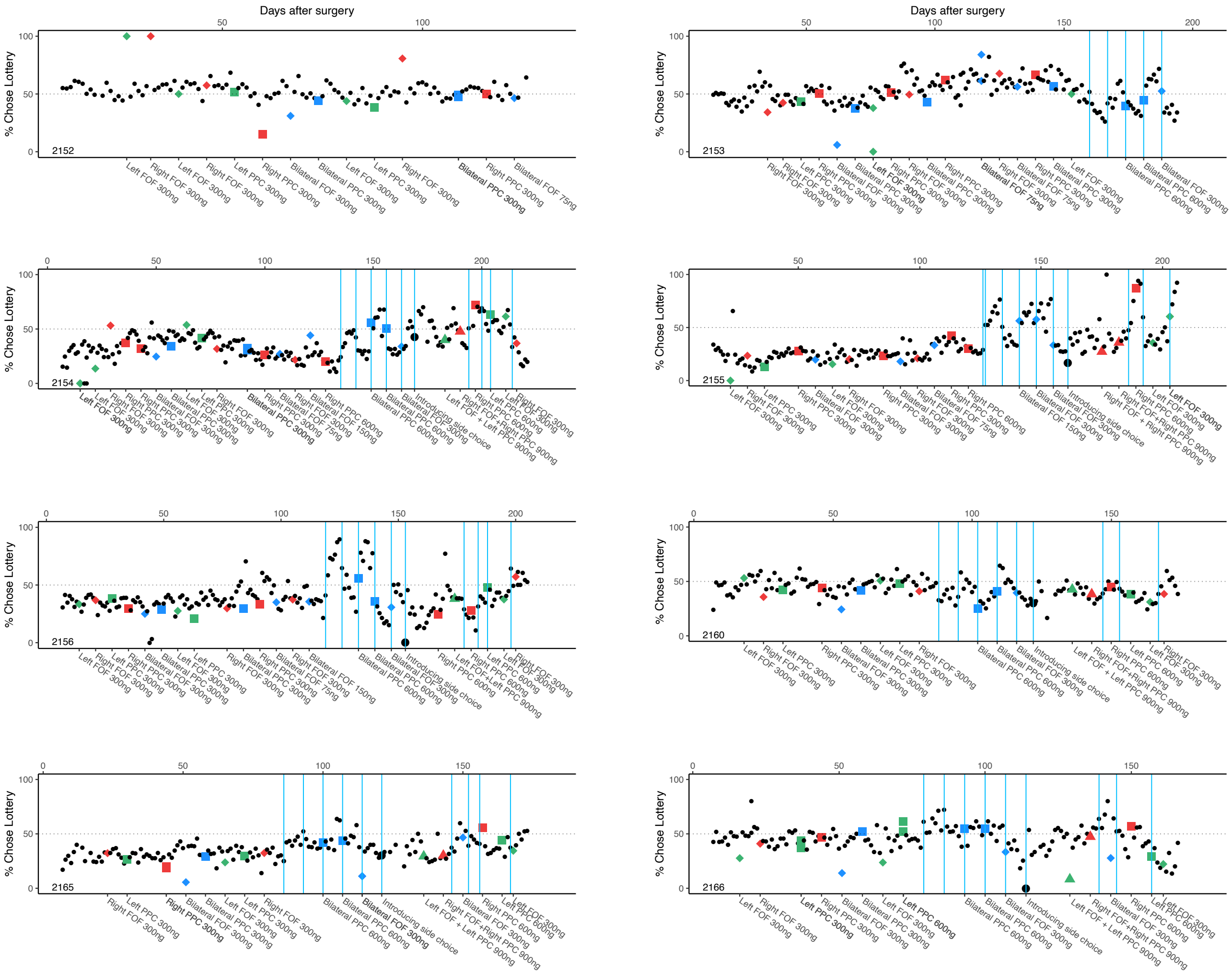

Figure 2. Timeline of muscimol experiments for each animal. Each point is the percentage choosing lottery for the given session. The number at the x-axis indicates the days passed since the surgical implantation of cannulae. Control days are shown as small black dots. Right infusions are shown in red, left infusions are in green, and bilateral infusions are shown in blue. FOF infusions are represented by diamonds, PPC infusions by squares, both FOF and PPC infusions by triangles. The blue bars indicate the day of a model-based surebet value change. The large black dot indicates the day when free choice trials were introduced. The bottom x-labels describe the details (side, region and dose) of each infusion. For bilateral and multi-region infusions, the dose is the total amount of muscimol. For example, '300 ng in bilateral FOF', means that 150ng were infused on each side.

1.3 Histology

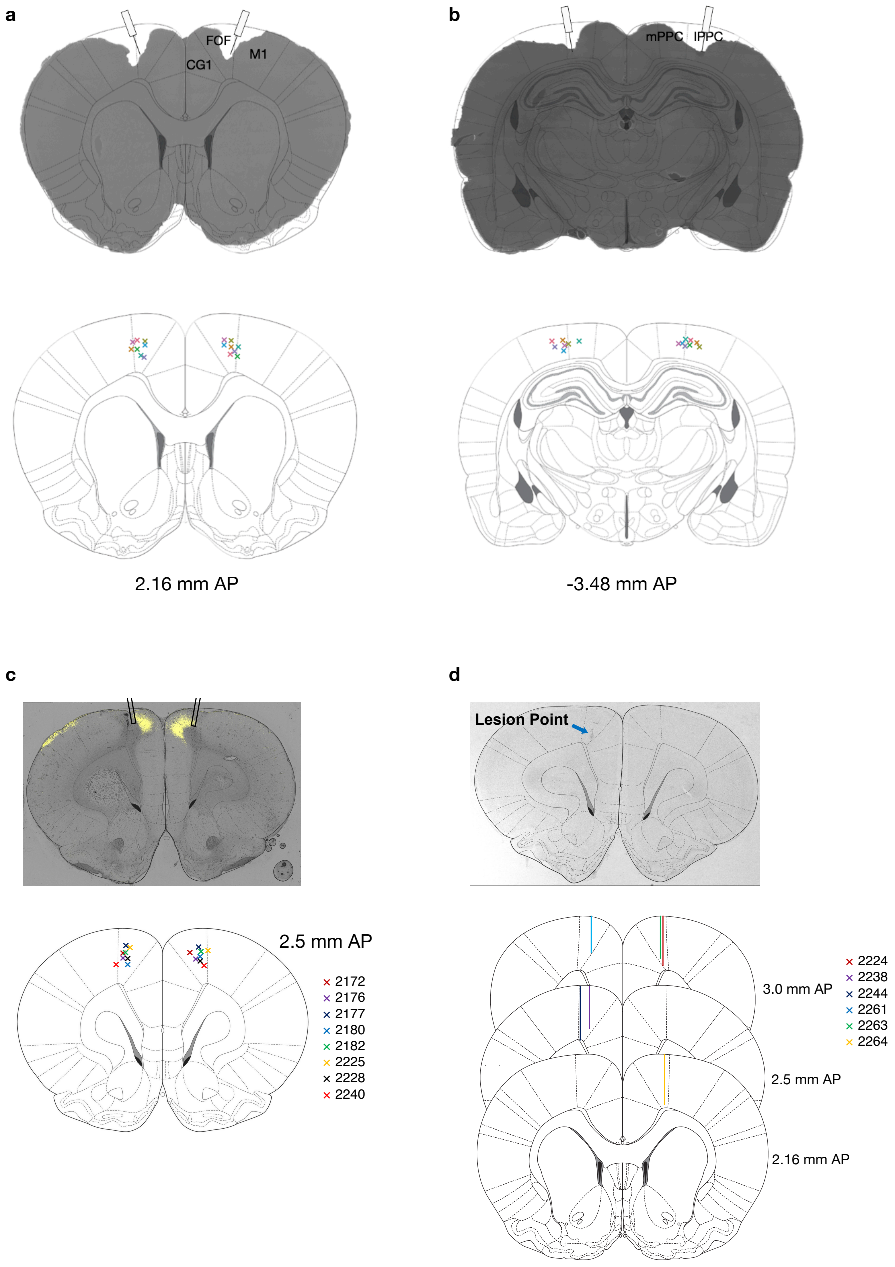

Figure 3. Histology. Each panel contains an example for that experiment (top) and a summary of the location of implanted hardware (bottom). **a.** FOF muscimol. Note, that in the nomenclature of Paxinos and Watson (2004) the area that we describe as the FOF is considered to be part of M2. CG1 = Cingulate Cortex; M1 = Primary motor cortex. (n = 8 rats) **b.** PPC muscimol. Coronal section of an example rat brain showing cannulae implanted at 10° in PPC, overlaid with a section 3.48 mm posterior to Bregma. mPPC = medial PPC, lPPC = lateral PPC. (n = 7 rats) **c.** FOF optical fibres. Note: the bright yellow indicates spread of virus which was unlikely to be photoactivated. The photoactivated region has been bleached by repeated laser stimulation. (n = 8 rats) **d.** FOF electrodes tracks. Recordings were performed along the entire length of the track. (n = 6 rats)

1.4 Influence of silencing FOF and PPC on Reaction times

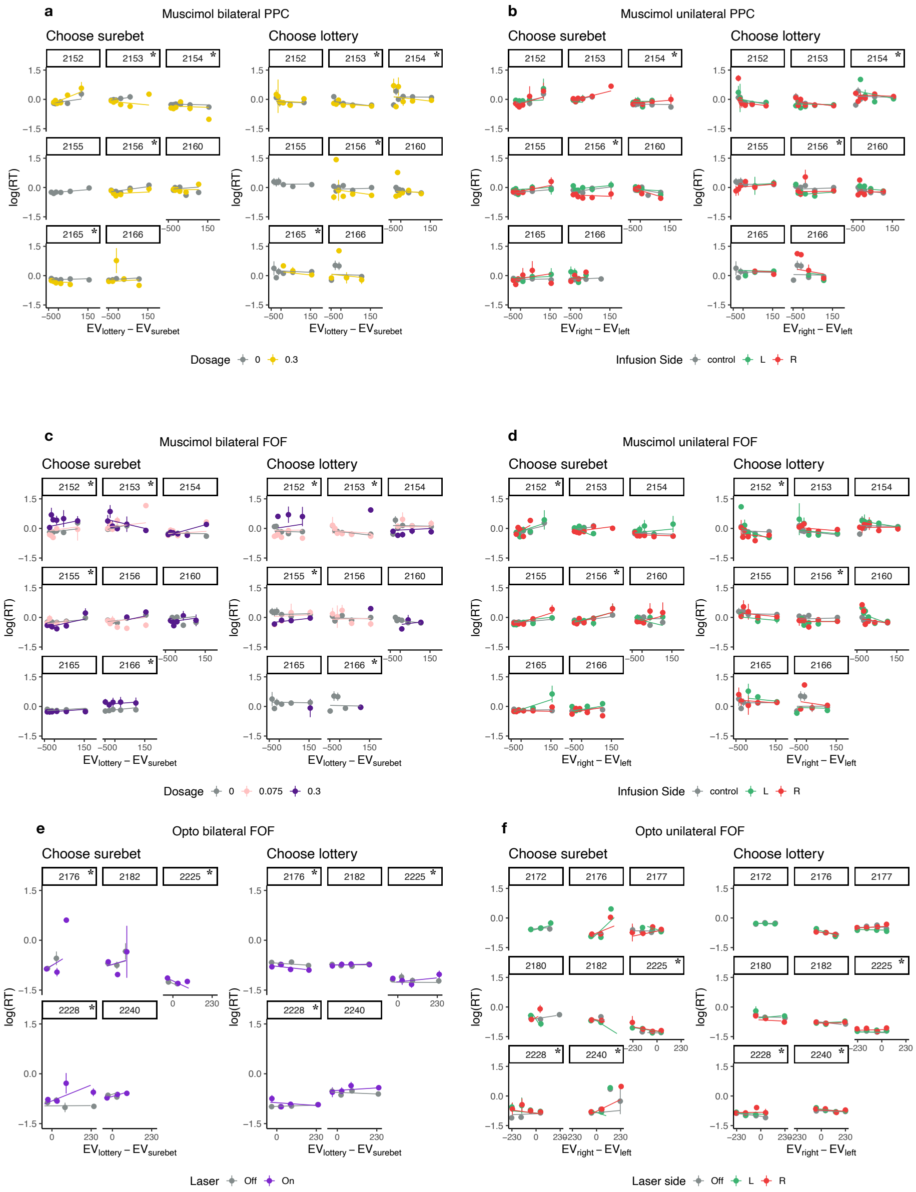

Figure 4. Reaction time (RT) and LMM model fits for bilateral FOF and PPC inactivations. The circles with error bars represent the mean and standard error of log(RT). The lines are the model predictions generated by the LMM. **a.** Bilateral PPC muscimol (n = 1036 trials, 7 rats). Reaction times are from the same trials as presented in Extended Data Fig. 2a. **b.** Unilateral PPC muscimol (n = 2450 trials, 8 rats). Reaction times are from the same trials as presented in Extended Data Fig. 2d. **c.** Bilateral FOF muscimol (n = 924 trials, 8 rats). Reaction times are from the same trials as presented in Extended Data Fig. 2b. **d.** Unilateral FOF muscimol (n = 2401 trials, 8 rats). Reaction times are from the same trials as presented in Extended Data Fig. 2e. **e.** Bilateral FOF optogenetic (n = 3058 trials, 5 rats). Reaction times are from the same trials as presented in Extended Data Fig. 2c. **f.** Unilateral FOF optogenetic (n = 13080 trials, 8 rats). Reaction times are from the same trials as presented in Extended Data Fig. 2f.

2 Supplementary Tables

2.1 Bilateral opto fits

		ρ	σ	$\omega_{rational}$	$\omega_{lottery}$	$\omega_{surebet}$
**2176**	Control	1.46 [1.28, 1.63]	0.02 [0.01, 0.03]	0.99 [0.97, 1.00]	0.01 [0.00, 0.02]	0.01 [0.00, 0.02]
	ΔBi FOF	-0.29 [-0.47, -0.10]	0.01 [-0.00, 0.01]	-0.01 [-0.04, 0.01]	0.00 [-0.01, 0.02]	0.01 [-0.01, 0.02]
**2182**	Control	1.19 [0.95, 1.53]	0.08 [0.04, 0.10]	0.98 [0.95, 1.00]	0.01 [0.00, 0.03]	0.01 [0.00, 0.03]
	ΔBi FOF	-0.24 [-0.42, -0.08]	0.02 [-0.00, 0.05]	-0.01 [-0.05, 0.01]	0.00 [-0.01, 0.03]	0.01 [-0.01, 0.03]
**2225**	Control	1.00 [0.75, 1.28]	0.05 [0.03, 0.07]	0.98 [0.96, 1.00]	0.01 [0.00, 0.03]	0.01 [0.00, 0.02]
	ΔBi FOF	-0.20 [-0.34, -0.07]	0.01 [-0.00, 0.03]	-0.01 [-0.05, 0.01]	0.00 [-0.01, 0.03]	0.01 [-0.01, 0.03]
**2228**	Control	0.83 [0.72, 0.97]	0.06 [0.05, 0.07]	0.98 [0.95, 0.99]	0.01 [0.00, 0.03]	0.01 [0.00, 0.03]
	ΔBi FOF	-0.17 [-0.27, -0.06]	0.02 [-0.00, 0.04]	-0.02 [-0.07, 0.01]	0.00 [-0.01, 0.04]	0.01 [-0.01, 0.05]
**2240**	Control	1.18 [0.96, 1.44]	0.04 [0.03, 0.06]	0.98 [0.96, 1.00]	0.01 [0.00, 0.02]	0.01 [0.00, 0.02]
	ΔBi FOF	-0.24 [-0.39, -0.08]	0.01 [-0.00, 0.03]	-0.01 [-0.04, 0.01]	0.00 [-0.01, 0.02]	0.01 [-0.01, 0.03]

Table 1. Fits from the 3-agent inactivation model for the bilateral fof optogenetic silencing experiment. The median of the parameter posterior distribution is reported along with its 95% confidence interval in brackets.

2.2 Unilateral opto fits

		ρ	σ	$\omega_{rational}$	$\omega_{lottery}$	$\omega_{surebet}$
**2172**	Control	0.78 [0.69, 0.98]	0.09 [0.06, 0.11]	0.97 [0.91, 0.98]	0.01 [0.00, 0.03]	0.03 [0.01, 0.07]
	ΔUni FOF	-0.08 [-0.14, -0.02]	0.01 [-0.01, 0.02]	-0.02 [-0.05, 0.00]	-0.00 [-0.02, 0.01]	0.02 [0.00, 0.05]
**2176**	Control	1.37 [1.25, 1.49]	0.02 [0.02, 0.03]	0.98 [0.96, 0.99]	0.01 [0.00, 0.02]	0.02 [0.01, 0.02]
	ΔUni FOF	-0.13 [-0.23, -0.04]	0.00 [-0.00, 0.01]	-0.01 [-0.03, 0.00]	-0.00 [-0.01, 0.01]	0.01 [0.00, 0.03]
**2177**	Control	0.96 [0.86, 1.09]	0.08 [0.06, 0.09]	0.97 [0.95, 0.99]	0.01 [0.00, 0.02]	0.02 [0.01, 0.04]
	ΔUni FOF	-0.09 [-0.16, -0.03]	0.01 [-0.00, 0.02]	-0.02 [-0.04, 0.00]	-0.00 [-0.01, 0.01]	0.02 [0.00, 0.04]
**2180**	Control	0.79 [0.68, 0.93]	0.05 [0.04, 0.07]	0.97 [0.95, 0.99]	0.01 [0.00, 0.02]	0.02 [0.01, 0.04]
	ΔUni FOF	-0.08 [-0.14, -0.02]	0.00 [-0.00, 0.01]	-0.01 [-0.03, 0.00]	-0.00 [-0.01, 0.01]	0.02 [0.00, 0.03]
**2182**	Control	1.02 [0.89, 1.18]	0.11 [0.09, 0.13]	0.98 [0.96, 0.99]	0.01 [0.00, 0.02]	0.02 [0.01, 0.03]
	ΔUni FOF	-0.10 [-0.17, -0.03]	0.01 [-0.01, 0.02]	-0.01 [-0.03, 0.00]	-0.00 [-0.01, 0.01]	0.01 [0.00, 0.03]
**2225**	Control	0.78 [0.71, 0.86]	0.06 [0.05, 0.06]	0.98 [0.96, 0.99]	0.01 [0.00, 0.02]	0.02 [0.01, 0.03]
	ΔUni FOF	-0.08 [-0.13, -0.02]	0.00 [-0.00, 0.01]	-0.01 [-0.03, 0.00]	-0.00 [-0.01, 0.01]	0.02 [0.00, 0.03]
**2228**	Control	0.63 [0.58, 0.69]	0.06 [0.05, 0.07]	0.97 [0.96, 0.99]	0.01 [0.00, 0.02]	0.02 [0.01, 0.03]
	ΔUni FOF	-0.06 [-0.11, -0.02]	0.00 [-0.00, 0.01]	-0.02 [-0.03, 0.00]	-0.00 [-0.01, 0.01]	0.02 [0.00, 0.03]
**2240**	Control	1.28 [1.14, 1.42]	0.04 [0.03, 0.05]	0.98 [0.96, 0.99]	0.01 [0.00, 0.02]	0.02 [0.01, 0.03]
	ΔUni FOF	-0.13 [-0.21, -0.04]	0.00 [-0.00, 0.01]	-0.01 [-0.03, 0.00]	-0.00 [-0.01, 0.01]	0.02 [0.00, 0.03]

Table 2. Fits from the 3-agent inactivation model for the unilateral fof optogenetic silencing experiment. The median of the parameter posterior distribution is reported along with its 95% confidence interval in brackets.

2.3 Muscimol fits

		ρ	σ	$\omega_{rational}$	$\omega_{lottery}$	$\omega_{surebet}$
	Control	0.64 [0.58, 0.71]	0.05 [0.04, 0.06]	0.84 [0.79, 0.88]	0.14 [0.10, 0.18]	0.03 [0.01, 0.04]
	ΔBi FOF	-0.10 [-0.27, 0.04]	0.03 [-0.01, 0.09]	-0.14 [-0.41, 0.17]	-0.09 [-0.15, -0.03]	0.23 [-0.03, 0.49]
**2152**	ΔUni FOF	-0.06 [-0.09, -0.02]	0.02 [0.00, 0.03]	-0.04 [-0.09, 0.01]	0.01 [-0.03, 0.04]	0.03 [0.01, 0.06]
	ΔBi PPC	-0.02 [-0.07, 0.03]	0.00 [-0.01, 0.02]	0.02 [-0.04, 0.07]	-0.05 [-0.09, -0.00]	0.03 [-0.00, 0.07]
	ΔUni PPC	0.01 [-0.02, 0.04]	0.00 [-0.01, 0.01]	-0.00 [-0.04, 0.04]	-0.03 [-0.06, 0.00]	0.03 [0.01, 0.05]
	Control	0.76 [0.67, 0.89]	0.05 [0.04, 0.07]	0.84 [0.78, 0.93]	0.15 [0.06, 0.21]	0.01 [0.00, 0.02]
	ΔBi FOF	-0.12 [-0.32, 0.05]	0.03 [-0.01, 0.09]	-0.13 [-0.40, 0.18]	-0.07 [-0.17, 0.04]	0.20 [-0.01, 0.46]
**2153**	ΔUni FOF	-0.06 [-0.10, -0.03]	0.02 [0.00, 0.03]	-0.04 [-0.08, 0.01]	0.01 [-0.02, 0.04]	0.03 [0.01, 0.06]
	ΔBi PPC	-0.03 [-0.08, 0.03]	0.01 [-0.01, 0.02]	0.02 [-0.03, 0.07]	-0.03 [-0.07, 0.01]	0.01 [0.00, 0.04]
	ΔUni PPC	0.01 [-0.02, 0.05]	0.00 [-0.01, 0.01]	-0.00 [-0.04, 0.03]	-0.01 [-0.04, 0.03]	0.01 [0.00, 0.02]
	Control	0.45 [0.42, 0.49]	0.06 [0.04, 0.08]	0.90 [0.83, 0.97]	0.04 [0.02, 0.06]	0.06 [0.00, 0.12]
	ΔBi FOF	-0.08 [-0.20, 0.03]	0.03 [-0.01, 0.10]	-0.09 [-0.31, 0.14]	-0.03 [-0.06, -0.01]	0.12 [-0.10, 0.34]
**2154**	ΔUni FOF	-0.04 [-0.06, -0.02]	0.02 [0.01, 0.04]	-0.02 [-0.05, 0.00]	-0.01 [-0.02, 0.01]	0.02 [0.00, 0.06]
	ΔBi PPC	-0.02 [-0.05, 0.02]	0.01 [-0.01, 0.02]	0.01 [-0.02, 0.05]	-0.02 [-0.04, -0.00]	0.01 [-0.02, 0.04]
	ΔUni PPC	0.01 [-0.01, 0.03]	0.00 [-0.01, 0.01]	0.00 [-0.02, 0.02]	-0.01 [-0.03, -0.00]	0.01 [-0.00, 0.04]
	Control	0.39 [0.36, 0.42]	0.05 [0.04, 0.06]	0.90 [0.78, 0.96]	0.05 [0.03, 0.07]	0.06 [0.01, 0.16]
	ΔBi FOF	-0.06 [-0.17, 0.03]	0.03 [-0.01, 0.08]	-0.08 [-0.29, 0.18]	-0.04 [-0.06, -0.01]	0.12 [-0.14, 0.33]
**2155**	ΔUni FOF	-0.03 [-0.05, -0.01]	0.02 [0.01, 0.03]	-0.03 [-0.08, 0.01]	-0.01 [-0.03, 0.01]	0.04 [0.01, 0.10]
	ΔBi PPC	no data	no data	no data	no data	no data
	ΔUni PPC	no data	no data	no data	no data	no data
	Control	0.47 [0.44, 0.50]	0.02 [0.01, 0.03]	0.74 [0.69, 0.78]	0.14 [0.12, 0.17]	0.12 [0.08, 0.16]
	ΔBi FOF	-0.08 [-0.20, 0.03]	0.01 [-0.00, 0.04]	-0.15 [-0.45, 0.25]	-0.12 [-0.15, -0.09]	0.27 [-0.13, 0.58]
**2156**	ΔUni FOF	-0.04 [-0.06, -0.02]	0.01 [0.00, 0.01]	-0.05 [-0.11, 0.01]	-0.03 [-0.06, 0.00]	0.08 [0.02, 0.14]
	ΔBi PPC	-0.02 [-0.05, 0.02]	0.00 [-0.00, 0.01]	0.02 [-0.05, 0.09]	-0.07 [-0.11, -0.03]	0.05 [-0.02, 0.12]
	ΔUni PPC	0.01 [-0.01, 0.03]	0.00 [-0.00, 0.00]	-0.00 [-0.05, 0.05]	-0.06 [-0.09, -0.03]	0.06 [0.01, 0.11]
	Control	0.60 [0.55, 0.66]	0.03 [0.02, 0.04]	0.78 [0.74, 0.83]	0.18 [0.14, 0.22]	0.03 [0.01, 0.06]
	ΔBi FOF	-0.10 [-0.25, 0.04]	0.02 [-0.01, 0.05]	-0.15 [-0.44, 0.21]	-0.13 [-0.19, -0.06]	0.28 [-0.03, 0.56]
**2160**	ΔUni FOF	-0.05 [-0.08, -0.02]	0.01 [0.00, 0.02]	-0.04 [-0.10, 0.01]	0.00 [-0.04, 0.04]	0.04 [0.01, 0.08]
	ΔBi PPC	-0.02 [-0.06, 0.02]	0.00 [-0.01, 0.01]	0.02 [-0.05, 0.08]	-0.05 [-0.10, -0.00]	0.03 [0.00, 0.08]
	ΔUni PPC	0.01 [-0.02, 0.04]	0.00 [-0.00, 0.01]	-0.00 [-0.04, 0.04]	-0.02 [-0.06, 0.01]	0.02 [0.01, 0.05]
	Control	0.45 [0.42, 0.48]	0.03 [0.01, 0.05]	0.89 [0.83, 0.94]	0.06 [0.03, 0.09]	0.05 [0.01, 0.11]
	ΔBi FOF	-0.07 [-0.19, 0.03]	0.02 [-0.01, 0.05]	-0.12 [-0.36, 0.12]	-0.05 [-0.07, -0.02]	0.17 [-0.07, 0.41]
**2165**	ΔUni FOF	-0.04 [-0.06, -0.02]	0.01 [0.00, 0.02]	-0.02 [-0.06, 0.01]	-0.01 [-0.03, 0.01]	0.03 [0.01, 0.07]
	ΔBi PPC	-0.02 [-0.05, 0.02]	0.00 [-0.01, 0.01]	0.01 [-0.02, 0.04]	-0.02 [-0.04, -0.00]	0.01 [-0.01, 0.04]
	ΔUni PPC	0.01 [-0.01, 0.03]	0.00 [-0.01, 0.01]	-0.00 [-0.03, 0.03]	-0.02 [-0.04, -0.01]	0.02 [-0.00, 0.05]
	Control	0.47 [0.42, 0.51]	0.04 [0.02, 0.05]	0.90 [0.84, 0.94]	0.08 [0.04, 0.12]	0.03 [0.00, 0.06]
	ΔBi FOF	-0.08 [-0.19, 0.03]	0.02 [-0.01, 0.07]	-0.12 [-0.35, 0.11]	-0.06 [-0.10, -0.02]	0.18 [-0.03, 0.41]
**2166**	ΔUni FOF	-0.04 [-0.06, -0.02]	0.01 [0.00, 0.02]	-0.02 [-0.06, 0.01]	-0.01 [-0.03, 0.01]	0.03 [0.01, 0.07]
	ΔBi PPC	-0.02 [-0.05, 0.02]	0.00 [-0.01, 0.02]	0.01 [-0.02, 0.04]	-0.02 [-0.05, 0.00]	0.01 [-0.00, 0.04]
	ΔUni PPC	0.01 [-0.01, 0.03]	0.00 [-0.01, 0.01]	-0.00 [-0.02, 0.02]	-0.01 [-0.03, 0.01]	0.01 [0.00, 0.03]

Table 3. Fits from the 3-agent inactivation model for the muscimol experiments. The median of the parameter posterior distribution is reported along with its 95% confidence interval in brackets.

2.4 Electrophysiology fits

subjid	ρ	σ	$\omega_{rational}$	$\omega_{lottery}$	$\omega_{surebet}$
2224	1.64 [1.33, 2.03]	0.02 [0.01, 0.03]	0.74 [0.66, 0.84]	0.19 [0.10, 0.28]	0.06 [0.04, 0.08]
2238	0.39 [0.34, 0.48]	0.07 [0.05, 0.11]	0.75 [0.68, 0.85]	0.15 [0.05, 0.20]	0.10 [0.07, 0.14]
2244	0.59 [0.52, 0.67]	0.06 [0.05, 0.07]	0.95 [0.92, 0.97]	0.03 [0.01, 0.05]	0.03 [0.01, 0.04]
2261	0.98 [0.86, 1.11]	0.03 [0.02, 0.04]	0.84 [0.79, 0.88]	0.01 [0.00, 0.03]	0.15 [0.11, 0.20]
2263	0.59 [0.44, 0.78]	0.17 [0.13, 0.21]	0.88 [0.80, 0.96]	0.03 [0.00, 0.08]	0.09 [0.03, 0.15]
2264	0.90 [0.78, 1.04]	0.05 [0.04, 0.06]	0.92 [0.88, 0.95]	0.02 [0.00, 0.05]	0.06 [0.04, 0.08]

Table 4. Fits from the 3-agent model for all electrophysiology recording animals. The median of the parameter posterior distribution is reported along with its 95% confidence interval in brackets.

2.5 Experimental Parameters

Experiment	Subjid	Sex	Sound	Cues	Mapping	Lottery Side	% win
Muscimol	2152	M	Pure tone	[2.49, 2.83, 3.23, 4.19, 7.04, 19.91] kHz	Positive	Right	55
Muscimol	2153	M	Pure tone	[2.49, 2.83, 3.23, 4.19, 7.04, 19.91] kHz	Positive	Right	50
Muscimol	2154	M	Pure tone	[2.49, 2.83, 3.23, 4.19, 7.04, 19.91] kHz	Positive	Right	50
Muscimol	2155	M	Pure tone	[2.49, 2.83, 3.23, 4.19, 7.04, 19.91] kHz	Positive	Right	50
Muscimol	2156	M	Pure tone	[2.49, 2.83, 3.23, 4.19, 7.04, 19.91] kHz	Positive	Right	50
Muscimol	2160	M	Pure tone	[2.49, 2.83, 3.23, 4.19, 7.04, 19.91] kHz	Positive	Left	50
Muscimol	2165	M	Pure tone	[2.49, 2.83, 3.23, 4.19, 7.04, 19.91] kHz	Positive	Right	50
Muscimol	2166	M	Pure tone	[2.49, 2.83, 3.23, 4.19, 7.04, 19.91] kHz	Positive	Right	60
Opto	2172	F	Clicks (10kHz)	[28, 45, 60, 81, 110, 151] Hz	Positive	Right	60
Opto	2176	M	Clicks (9.5kHz)	[28, 45, 60, 81, 110, 151] Hz	Positive	Right	55
Opto	2177	M	Clicks (9.5kHz)	[28, 45, 60, 81, 110, 151] Hz	Positive	Left	55
Opto	2180	M	Clicks (10kHz)	[28, 45, 60, 81, 110, 151] Hz	Positive	Right	60
Opto	2182	M	Clicks (10kHz)	[212, 195, 180, 159, 130, 89] Hz	Negative	Right	60
Opto	2225	F	Clicks (11kHz)	[28, 45, 60, 81, 110, 151] Hz	Positive	Left	70
Opto	2228	F	Clicks (11kHz)	[28, 45, 60, 81, 110, 151] Hz	Positive	Left	70
Opto	2240	M	Clicks (11kHz)	[28, 45, 60, 81, 110, 151] Hz	Positive	Right	70
Ephys	2224	F	Clicks (10kHz)	[28, 45, 60, 81, 110, 151] Hz	Positive	Left	60
Ephys	2244	M	Clicks (11.5kHz)	[28, 45, 60, 81, 110, 151] Hz	Positive	Right	75
Ephys	2238	M	Clicks (11KHz)	[212, 195, 180, 159, 130, 89] Hz	Negative	Right	70
Ephys	2261	M	Clicks (9kHz)	[28, 45, 60, 81, 110, 151] Hz	Positive	Left (ipsi)	50
Ephys	2263	M	Clicks (11KHz)	[212, 195, 180, 159, 130, 89] Hz	Negative	Left	70
Ephys	2264	M	Clicks (11kHz)	[28, 45, 60, 81, 110, 151] Hz	Positive	Right (ipsi)	70

Table 5. Details of the experimental parameters for all subjects. In the for animals that were trained with click trains, the pitch of each click was set based on the % win and is indicated in parenthesis. 'Positive' mapping means that higher frequencies (either clicks or pure tones) were mapped to higher lottery magnitudes and 'negative' mapping means that lower frequencies were mapped to higher lottery magnitudes. For the two animals with 'ipsi' noted in the 'Lottery Side' columns, the neural activity was recorded on the same side as the lottery port. For the other animals recordings were contralateral to the lottery port.

3 Statistical Appendix

3.1 Figure 2b: Muscimol bilateral PPC

3.1.1 Model Summary

```
Generalized linear mixed model fit by maximum likelihood (Laplace
  Approximation) [glmerMod]
 Family: binomial  ( logit )
Formula: choice ~ delta_ev * dosage + (dosage * delta_ev | subjid)
   Data: m_bi_ppc_df

     AIC      BIC   logLik deviance df.resid
  7615.0   7715.2  -3793.5   7587.0     9487

Scaled residuals:
     Min       1Q   Median       3Q      Max
-20.4740  -0.4716  -0.2671   0.2548   5.6187

Random effects:
 Groups Name            Variance  Std.Dev. Corr
 subjid (Intercept)     0.5588627 0.74757
        dosage          1.0107657 1.00537  -0.86
        delta_ev        0.0001436 0.01198   0.74 -0.29
        dosage:delta_ev 0.0003095 0.01759  -0.86  0.48 -0.98
Number of obs: 9501, groups:  subjid, 8

Fixed effects:
                 Estimate Std. Error z value Pr(>|z|)
(Intercept)     -1.225506   0.268004  -4.573 4.81e-06 ***
delta_ev         0.040412   0.004377   9.232  < 2e-16 ***
dosage          -0.603039   0.577524  -1.044    0.296
delta_ev:dosage -0.018572   0.011588  -1.603    0.109
---
Signif. codes:  0 '***' 0.001 '**' 0.01 '*' 0.05 '.' 0.1 ' ' 1

Correlation of Fixed Effects:
            (Intr) delt_v dosage
delta_ev     0.695
dosage      -0.560 -0.159
delta_v:dsg -0.444 -0.578 -0.116
optimizer (Nelder_Mead) convergence code: 0 (OK)
```

3.1.2 Likelihood Ratio Test

```
Likelihood ratio test

Model 1: choice ~ delta_ev * dosage + (dosage * delta_ev | subjid)
Model 2: choice ~ delta_ev + (dosage * delta_ev | subjid)
  #Df  LogLik Df  Chisq Pr(>Chisq)
1  14 -3793.5
2  12 -3795.1 -2 3.1507     0.2069
```

3.2 Figure 2c: Muscimol bilateral FOF

3.2.1 Model Summary

```
Generalized linear mixed model fit by maximum likelihood (Laplace
  Approximation) [glmerMod]
 Family: binomial  ( logit )
Formula: choice ~ delta_ev * dosage + (delta_ev * dosage | subjid)
   Data: m_bi_fof_df

     AIC      BIC   logLik deviance df.resid
  7513.7   7613.7  -3742.8   7485.7     9375

Scaled residuals:
     Min       1Q   Median       3Q      Max
-18.7301  -0.4709  -0.2409   0.2562   5.6846

Random effects:
 Groups Name            Variance  Std.Dev. Corr
 subjid (Intercept)     0.5633506 0.75057
        delta_ev        0.0001397 0.01182   0.73
        dosage          2.4487507 1.56485  -0.32 -0.85
        delta_ev:dosage 0.0020130 0.04487  -0.86 -0.77  0.60
Number of obs: 9389, groups:  subjid, 8

Fixed effects:
                 Estimate Std. Error z value Pr(>|z|)
(Intercept)     -1.223594   0.268284  -4.561 5.10e-06 ***
delta_ev         0.040173   0.004311   9.318  < 2e-16 ***
dosage          -3.196055   0.933027  -3.425 0.000614 ***
delta_ev:dosage -0.076208   0.018075  -4.216 2.48e-05 ***
---
Signif. codes:  0 '***' 0.001 '**' 0.01 '*' 0.05 '.' 0.1 ' ' 1

Correlation of Fixed Effects:
            (Intr) delt_v dosage
delta_ev     0.681
dosage      -0.211 -0.483
delta_v:dsg -0.730 -0.702  0.107
optimizer (Nelder_Mead) convergence code: 0 (OK)
```

3.2.2 Likelihood Ratio Test

```
Likelihood ratio test

Model 1: choice ~ delta_ev * dosage + (delta_ev * dosage | subjid)
Model 2: choice ~ delta_ev + (delta_ev * dosage | subjid)
  #Df  LogLik Df Chisq Pr(>Chisq)
1  14 -3742.8
2  12 -3751.0 -2 16.43  0.0002706 ***
---
Signif. codes:  0 '***' 0.001 '**' 0.01 '*' 0.05 '.' 0.1 ' ' 1
```

3.3 Figure 2d: Opto bilateral FOF

3.3.1 Model Summary

```
Generalized linear mixed model fit by maximum likelihood (Laplace
  Approximation) [glmerMod]
 Family: binomial  ( logit )
Formula: choice ~ delta_ev * opto_contr_ipsi + (delta_ev * opto_contr_ipsi |
    sessid)
   Data: o_bi_fof_df

     AIC      BIC   logLik deviance df.resid
  2105.5   2189.9  -1038.8   2077.5     3044

Scaled residuals:
    Min      1Q  Median      3Q     Max
-94.152  -0.402   0.001   0.224   5.116

Random effects:
 Groups Name                            Variance  Std.Dev. Corr
 sessid (Intercept)                     0.6813040 0.82541
        delta_ev                        0.0008891 0.02982   0.65
        opto_contr_ipsibilateral        0.3119588 0.55853  -0.44 -0.42
        delta_ev:opto_contr_ipsibilateral 0.0006669 0.02582   0.35 -0.44 -0.13
Number of obs: 3058, groups:  sessid, 29

Fixed effects:
                                   Estimate Std. Error z value Pr(>|z|)
(Intercept)                        0.448214   0.183595   2.441 0.014634 *
delta_ev                           0.072866   0.007048  10.339  < 2e-16 ***
opto_contr_ipsibilateral          -0.628303   0.181463  -3.462 0.000535 ***
delta_ev:opto_contr_ipsibilateral -0.011915   0.008087  -1.473 0.140638
---
Signif. codes:  0 '***' 0.001 '**' 0.01 '*' 0.05 '.' 0.1 ' ' 1

Correlation of Fixed Effects:
            (Intr) delt_v opt_c_
delta_ev     0.626
opt_cntr_ps -0.494 -0.380
dlt_v:pt_c_  0.024 -0.526  0.217
optimizer (Nelder_Mead) convergence code: 0 (OK)
```

3.3.2 Likelihood Ratio Test

```
Likelihood ratio test

Model 1: choice ~ delta_ev * opto_contr_ipsi + (delta_ev * opto_contr_ipsi |
    sessid)
Model 2: choice ~ delta_ev + (delta_ev * opto_contr_ipsi | sessid)
  #Df  LogLik Df  Chisq Pr(>Chisq)
1  14 -1038.8
2  12 -1044.5 -2 11.421   0.003312 **
---
```

```
Signif. codes:  0 '***' 0.001 '**' 0.01 '*' 0.05 '.' 0.1 ' ' 1
```

3.4 Figure 2e: Muscimol unilateral PPC

3.4.1 Model Summary

```
Generalized linear mixed model fit by maximum likelihood (Laplace
  Approximation) [glmerMod]
 Family: binomial  ( logit )
Formula: choice ~ delta_ev * drug + (delta_ev * drug | subjid)
   Data: m_uni_ppc_mixS_df

     AIC      BIC   logLik deviance df.resid
  8828.5   8931.0  -4400.3   8800.5    11096

Scaled residuals:
     Min       1Q   Median       3Q      Max
-28.5928  -0.4644  -0.2572   0.2508   5.3816

Random effects:
 Groups Name                 Variance  Std.Dev. Corr
 subjid (Intercept)          5.414e-01 0.735798
        delta_ev             1.540e-04 0.012410  0.77
        drugmuscimol         2.508e-02 0.158367 -0.61 -0.51
        delta_ev:drugmuscimol 4.197e-06 0.002049 -0.39 -0.09  0.89
Number of obs: 11110, groups:  subjid, 8

Fixed effects:
                       Estimate Std. Error z value Pr(>|z|)
(Intercept)           -1.218459   0.263826  -4.618 3.87e-06 ***
delta_ev               0.040441   0.004525   8.936  < 2e-16 ***
drugmuscimol          -0.173003   0.095711  -1.808   0.0707 .
delta_ev:drugmuscimol -0.002300   0.002121  -1.085   0.2781
---
Signif. codes:  0 '***' 0.001 '**' 0.01 '*' 0.05 '.' 0.1 ' ' 1

Correlation of Fixed Effects:
            (Intr) delt_v drgmsc
delta_ev     0.726
drugmusciml -0.409 -0.271
dlt_v:drgms -0.111 -0.141 -0.026
optimizer (Nelder_Mead) convergence code: 0 (OK)
```

3.4.2 Likelihood Ratio Test

```
Likelihood ratio test

Model 1: choice ~ delta_ev * drug + (delta_ev * drug | subjid)
Model 2: choice ~ delta_ev + (delta_ev * drug | subjid)
  #Df  LogLik Df  Chisq Pr(>Chisq)
1  14 -4400.3
2  12 -4402.1 -2 3.6701     0.1596
```

3.5 Figure 2f: Muscimol unilateral FOF

3.5.1 Model Summary

```
Generalized linear mixed model fit by maximum likelihood (Laplace
  Approximation) [glmerMod]
 Family: binomial  ( logit )
Formula: choice ~ delta_ev * drug + (delta_ev * drug | subjid)
   Data: m_uni_fof_mixS_df

     AIC      BIC   logLik deviance df.resid
  8926.3   9028.4  -4449.2   8898.3    10852

Scaled residuals:
     Min       1Q   Median       3Q      Max
-23.8049  -0.4870  -0.2781   0.3169   5.5488

Random effects:
 Groups Name                  Variance  Std.Dev. Corr
 subjid (Intercept)           5.500e-01 0.741624
        delta_ev              1.541e-04 0.012412  0.73
        drugmuscimol          1.074e-01 0.327761 -0.41 -0.10
        delta_ev:drugmuscimol 3.183e-05 0.005641 -0.66 -0.88  0.50
Number of obs: 10866, groups:  subjid, 8

Fixed effects:
                       Estimate Std. Error z value Pr(>|z|)
(Intercept)           -1.228730   0.265455  -4.629 3.68e-06 ***
delta_ev               0.040443   0.004522   8.944  < 2e-16 ***
drugmuscimol          -0.076344   0.141001  -0.541    0.588
delta_ev:drugmuscimol -0.013323   0.002626  -5.074 3.90e-07 ***
---
Signif. codes:  0 '***' 0.001 '**' 0.01 '*' 0.05 '.' 0.1 ' ' 1

Correlation of Fixed Effects:
            (Intr) delt_v drgmsc
delta_ev     0.689
drugmusciml -0.374 -0.059
dlt_v:drgms -0.474 -0.748  0.195
optimizer (Nelder_Mead) convergence code: 0 (OK)
```

3.5.2 Likelihood Ratio Test

```
Likelihood ratio test

Model 1: choice ~ delta_ev * drug + (delta_ev * drug | subjid)
Model 2: choice ~ delta_ev + (delta_ev * drug | subjid)
  #Df  LogLik Df Chisq Pr(>Chisq)
1  14 -4449.2
2  12 -4455.9 -2 13.52   0.001159 **
---
Signif. codes:  0 '***' 0.001 '**' 0.01 '*' 0.05 '.' 0.1 ' ' 1
```

3.6 Figure 2g: Opto unilateral FOF

3.6.1 Model Summary

```
Generalized linear mixed model fit by maximum likelihood (Laplace
  Approximation) [glmerMod]
 Family: binomial  ( logit )
Formula: choice ~ delta_ev * isopto + (delta_ev * isopto | sessid)
   Data: o_uni_fof_mixS_df

     AIC      BIC   logLik deviance df.resid
  9725.9   9830.6  -4848.9   9697.9    13066

Scaled residuals:
    Min      1Q  Median      3Q     Max
-53.354  -0.438   0.001   0.303   4.424

Random effects:
 Groups Name             Variance  Std.Dev. Corr
 sessid (Intercept)      0.4188454 0.64718
        delta_ev         0.0004542 0.02131   0.11
        isopto1          0.0455323 0.21338   0.21 -0.74
        delta_ev:isopto1 0.0003477 0.01865   0.09 -0.52  0.56
Number of obs: 13080, groups:  sessid, 126

Fixed effects:
                  Estimate Std. Error z value Pr(>|z|)
(Intercept)      -0.126136   0.068914  -1.830   0.0672 .
delta_ev          0.055224   0.002478  22.286  < 2e-16 ***
isopto1          -0.323422   0.063402  -5.101 3.38e-07 ***
delta_ev:isopto1 -0.006952   0.002986  -2.328   0.0199 *
---
Signif. codes:  0 '***' 0.001 '**' 0.01 '*' 0.05 '.' 0.1 ' ' 1

Correlation of Fixed Effects:
            (Intr) delt_v isopt1
delta_ev     0.164
isopto1     -0.252 -0.278
delt_v:spt1 -0.031 -0.554  0.221
optimizer (Nelder_Mead) convergence code: 0 (OK)
```

3.6.2 Likelihood Ratio Test

```
Likelihood ratio test

Model 1: choice ~ delta_ev * isopto + (delta_ev * isopto | sessid)
Model 2: choice ~ delta_ev + (delta_ev * isopto | sessid)
  #Df  LogLik Df  Chisq Pr(>Chisq)
1  14 -4848.9
2  12 -4861.8 -2 25.839  2.449e-06 ***
---
Signif. codes:  0 '***' 0.001 '**' 0.01 '*' 0.05 '.' 0.1 ' ' 1
```

3.7 Figure 2h: Muscimol unilateral PPC (left vs. right)

3.7.1 Model Summary

```
Generalized linear mixed model fit by maximum likelihood (Adaptive
  Gauss-Hermite Quadrature, nAGQ = 0) [glmerMod]
 Family: binomial  ( logit )
Formula: choose_right ~ pro_right_delta_ev + region + (pro_right_delta_ev +
    region | subjid)
   Data: m_uni_ppc_mixS_df

     AIC      BIC   logLik deviance df.resid
  2085.6   2138.5  -1033.8   2067.6     2636

Scaled residuals:
    Min      1Q  Median      3Q     Max
-36.475  -0.423  -0.248   0.321  21.344

Random effects:
 Groups Name               Variance  Std.Dev. Corr
 subjid (Intercept)        1.3934326 1.18044
        pro_right_delta_ev 0.0001964 0.01401   0.79
        regionRight PPC    0.7744559 0.88003  -0.53 -0.04
Number of obs: 2645, groups:  subjid, 8

Fixed effects:
                    Estimate Std. Error z value Pr(>|z|)
(Intercept)        -1.457650   0.435015  -3.351 0.000806 ***
pro_right_delta_ev  0.038703   0.005231   7.398 1.38e-13 ***
regionRight PPC     0.383008   0.345989   1.107 0.268296
---
Signif. codes:  0 '***' 0.001 '**' 0.01 '*' 0.05 '.' 0.1 ' ' 1

Correlation of Fixed Effects:
            (Intr) pr_r__
pr_rght_dl_  0.702
regnRghtPPC -0.548 -0.018
```

3.7.2 Likelihood Ratio Test

```
Likelihood ratio test

Model 1: choose_right ~ pro_right_delta_ev + region + (pro_right_delta_ev +
    region | subjid)
Model 2: choose_right ~ pro_right_delta_ev + (pro_right_delta_ev + region |
    subjid)
  #Df  LogLik Df  Chisq Pr(>Chisq)
1   9 -1033.8
2   8 -1034.4 -1 1.1782     0.2777
```

3.8 Figure 2i: Muscimol unilateral FOF (left vs. right)

3.8.1 Model Summary

```
Generalized linear mixed model fit by maximum likelihood (Adaptive
  Gauss-Hermite Quadrature, nAGQ = 0) [glmerMod]
 Family: binomial  ( logit )
Formula: choose_right ~ pro_right_delta_ev + region + (pro_right_delta_ev +
    region | subjid)
   Data: m_uni_fof_mixS_df

     AIC      BIC   logLik deviance df.resid
  2162.9   2215.0  -1072.4   2144.9     2392

Scaled residuals:
    Min      1Q  Median      3Q     Max
-9.0742 -0.4908 -0.2731  0.4821  9.0394

Random effects:
 Groups Name               Variance  Std.Dev. Corr
 subjid (Intercept)        1.2694211 1.12669
        pro_right_delta_ev 0.0001033 0.01017   0.71
        regionRight FOF    0.3029769 0.55043  -0.33  0.44
Number of obs: 2401, groups:  subjid, 8

Fixed effects:
                    Estimate Std. Error z value Pr(>|z|)
(Intercept)        -1.521648   0.413500  -3.680 0.000233 ***
pro_right_delta_ev  0.028235   0.003799   7.433 1.06e-13 ***
regionRight FOF     0.694406   0.232811   2.983 0.002857 **
---
Signif. codes:  0 '***' 0.001 '**' 0.01 '*' 0.05 '.' 0.1 ' ' 1

Correlation of Fixed Effects:
            (Intr) pr_r__
pr_rght_dl_  0.615
regnRghtFOF -0.384  0.377
optimizer (bobyqa) convergence code: 0 (OK)
```

3.8.2 Likelihood Ratio Test

```
Likelihood ratio test

Model 1: choose_right ~ pro_right_delta_ev + region + (pro_right_delta_ev +
    region | subjid)
Model 2: choose_right ~ pro_right_delta_ev + (pro_right_delta_ev + region |
    subjid)
  #Df  LogLik Df  Chisq Pr(>Chisq)
1   9 -1072.5
2   8 -1075.8 -1 6.6332    0.01001 *
---
Signif. codes:  0 '***' 0.001 '**' 0.01 '*' 0.05 '.' 0.1 ' ' 1
```

3.9 Figure 2j: Opto unilateral FOF (left vs. right)

3.9.1 Model Summary

```
Generalized linear mixed model fit by maximum likelihood (Adaptive
  Gauss-Hermite Quadrature, nAGQ = 0) [glmerMod]
 Family: binomial  ( logit )
Formula: choose_right ~ pro_right_delta_ev + opto_out + (pro_right_delta_ev +
    opto_out | sessid)
   Data: o_uni_fof_mixS_df

     AIC      BIC   logLik deviance df.resid
  3653.2   3711.2  -1817.6   3635.2     4649

Scaled residuals:
     Min       1Q   Median       3Q      Max
-13.8803  -0.4418   0.0150   0.4039  25.1797

Random effects:
 Groups Name               Variance  Std.Dev. Corr
 sessid (Intercept)        0.5852412 0.76501
        pro_right_delta_ev 0.0004643 0.02155   0.09
        opto_outopto_right 0.4858004 0.69699  -0.36 -0.10
Number of obs: 4658, groups:  sessid, 126

Fixed effects:
                   Estimate Std. Error z value Pr(>|z|)
(Intercept)        0.051304   0.106683   0.481   0.6306
pro_right_delta_ev 0.045346   0.002467  18.384   <2e-16 ***
opto_outopto_right 0.373697   0.173146   2.158   0.0309 *
---
Signif. codes:  0 '***' 0.001 '**' 0.01 '*' 0.05 '.' 0.1 ' ' 1

Correlation of Fixed Effects:
            (Intr) pr_r__
pr_rght_dl_  0.049
opt_tpt_rgh -0.615  0.002
```

3.9.2 Likelihood Ratio Test

```
Likelihood ratio test

Model 1: choose_right ~ pro_right_delta_ev + opto_out + (pro_right_delta_ev +
    opto_out | sessid)
Model 2: choose_right ~ pro_right_delta_ev + (pro_right_delta_ev + opto_out |
    sessid)
  #Df  LogLik Df  Chisq Pr(>Chisq)
1   9 -1817.6
2   8 -1819.9 -1 4.5869    0.03222 *
---
Signif. codes:  0 '***' 0.001 '**' 0.01 '*' 0.05 '.' 0.1 ' ' 1
```
