# Supplementary material for: The rat frontal orienting field dynamically encodes value for economic decisions under risk
Source: Nat Neurosci. 2023 Oct 19;26(11):1942–52. doi: 10.1038/s41593-023-01461-x (PMC10620098; doi:10.1038/s41593-023-01461-x)
Supplement: Supplementary file 2 — Reporting Summary [file 41593_2023_1461_MOESM2_ESM.pdf]

Corresponding author(s): Jeffrey C. Erlich
Last updated by author(s): Aug 22, 2023

Reporting Summary

Nature Portfolio wishes to improve the reproducibility of the work that we publish. This form provides structure for consistency and transparency in reporting. For further information on Nature Portfolio policies, see our Editorial Policies and the Editorial Policy Checklist.

Statistics

For all statistical analyses, confirm that the following items are present in the figure legend, table legend, main text, or Methods section.

n/a	Confirmed	
☐	☒	The exact sample size (*n*) for each experimental group/condition, given as a discrete number and unit of measurement
☐	☒	A statement on whether measurements were taken from distinct samples or whether the same sample was measured repeatedly
☐	☒	The statistical test(s) used AND whether they are one- or two-sided<br>*Only common tests should be described solely by name; describe more complex techniques in the Methods section.*
☐	☒	A description of all covariates tested
☐	☒	A description of any assumptions or corrections, such as tests of normality and adjustment for multiple comparisons
☐	☒	A full description of the statistical parameters including central tendency (e.g. means) or other basic estimates (e.g. regression coefficient) AND variation (e.g. standard deviation) or associated estimates of uncertainty (e.g. confidence intervals)
☐	☒	For null hypothesis testing, the test statistic (e.g. *F*, *t*, *r*) with confidence intervals, effect sizes, degrees of freedom and *P* value noted<br>*Give P values as exact values whenever suitable.*
☐	☒	For Bayesian analysis, information on the choice of priors and Markov chain Monte Carlo settings
☐	☒	For hierarchical and complex designs, identification of the appropriate level for tests and full reporting of outcomes
☐	☒	Estimates of effect sizes (e.g. Cohen's *d*, Pearson's *r*), indicating how they were calculated

Our web collection on statistics for biologists contains articles on many of the points above.

Software and code

Policy information about availability of computer code

Data collection	Matlab 2018b, Bpod 0.5 and Psychtoolbox3 (for behavior control and acquisition). Open Ephys 0.5.5.1 (for in vivo extracellular electrophysiology acquisition).
Data analysis	Matlab 2022a, R 4.2.1, Kilosort 2, Phy 2.0a1, ImageJ 1.54f, brms 2.17.0, lme4 1.1-29, rstan 2.21.5, Julia 1.9.

For manuscripts utilizing custom algorithms or software that are central to the research but not yet described in published literature, software must be made available to editors and reviewers. We strongly encourage code deposition in a community repository (e.g. GitHub). See the Nature Portfolio guidelines for submitting code & software for further information.

Data

Policy information about availability of data

All manuscripts must include a data availability statement. This statement should provide the following information, where applicable:

- Accession codes, unique identifiers, or web links for publicly available datasets
- A description of any restrictions on data availability
- For clinical datasets or third party data, please ensure that the statement adheres to our policy

Code and Data is available at https://github.com/erlichlab/risk-fof-ppc-2023

Human research participants

Policy information about studies involving human research participants and Sex and Gender in Research.

Reporting on sex and gender	Not applicable
Population characteristics	Not applicable
Recruitment	Not applicable
Ethics oversight	Not applicable

Note that full information on the approval of the study protocol must also be provided in the manuscript.

Field-specific reporting

Please select the one below that is the best fit for your research. If you are not sure, read the appropriate sections before making your selection.

☒ Life sciences ☐ Behavioural & social sciences ☐ Ecological, evolutionary & environmental sciences

For a reference copy of the document with all sections, see nature.com/documents/nr-reporting-summary-flat.pdf

Life sciences study design

All studies must disclose on these points even when the disclosure is negative.

Sample size	No statistical methods were used to pre-determine sample sizes but our sample sizes are similar to those reported in previous publications (Hanks et al., 2015, Nature, DOI:10.1038/nature14066).
Data exclusions	As stated in the text, for the infusion experiments, we only included behavioral data from around the time of the infusions. This was done to avoid any possibility of misattributing the effects of inactivations to slow changes in behavior over time. As stated in the text, for some experiments, we used a "pre-screening" criteria (e.g. the sure-bet shifting experiment).
Replication	We used muscimol infusion and optogenetic inhibition to perturbate the FOF area during the risky decision-making task, both perturbation methods came to the consistent results. For the behavior data analysis, we used 'Rstan' to fit the individual behavior data, and used 'brms' to fit the population data, both methods gave us the consistent results. Electrophysiological data were collected from 6 rats by 2 researchers, and all the animals gave the consistent results.
Randomization	We used a within-subject design.
Blinding	All behavior data collection is computerized. Animals were placed in training rigs by a technician who was unaware of the overall goals of the experiment.

Reporting for specific materials, systems and methods

We require information from authors about some types of materials, experimental systems and methods used in many studies. Here, indicate whether each material, system or method listed is relevant to your study. If you are not sure if a list item applies to your research, read the appropriate section before selecting a response.

Materials & experimental systems

n/a	Involved in the study	
☒	☐	Antibodies
☒	☐	Eukaryotic cell lines
☒	☐	Palaeontology and archaeology
☐	☒	Animals and other organisms
☒	☐	Clinical data
☒	☐	Dual use research of concern

Methods

n/a	Involved in the study	
☒	☐	ChIP-seq
☒	☐	Flow cytometry
☒	☐	MRI-based neuroimaging

Animals and other research organisms

Policy information about studies involving animals; ARRIVE guidelines recommended for reporting animal research, and Sex and Gender in Research

Laboratory animals	A total of 26 rats (22 males, 4 female, between the age of 2 and 18 months) were used in this study, including 24 Sprague Dawley rats and 2 male Brown Norway rats (Vital River, Beijing, China).
Wild animals	No wild animals were used in this study.
Reporting on sex	Our lab shifted from performing experiments only on male rats to performing studies on both sexes. However, the data collected here was mostly from before the shift, which is why there was only 4 female in the study and thus we did not examine sex related differences.
Field-collected samples	No field-collected samples were used in this study.
Ethics oversight	Animal use procedures were approved by New York University Shanghai International Animal Care and Use Committee following both US and Chinese regulations.

Note that full information on the approval of the study protocol must also be provided in the manuscript.
